# The Enhanced Photocatalytic Sterilization of a Metal–Organic‐Framework‐Based S‐Scheme Heterostructure of Ag_2_S@PB for Rapid Healing of Bacteria‐Infected Open Wounds

**DOI:** 10.1002/smsc.202300114

**Published:** 2023-11-20

**Authors:** Zhenxing Yang, Cuihong Chen, Chaofeng Wang, Yufeng Zheng, Shuilin Wu, Yu Zhang, Xiangmei Liu

**Affiliations:** ^1^ Biomedical Materials Engineering Research Center Hubei Key Laboratory of Polymer Materials Ministry-of-Education Key Laboratory for the Green Preparation and Application of Functional Materials School of Materials Science & Engineering Hubei University Wuhan 430062 China; ^2^ School of Health Science & Biomedical Engineering Hebei University of Technology Xiping Avenue 5340# Tianjin 300401 China; ^3^ School of Materials Science & Engineering Peking University Yiheyuan Road 5# Beijing 100871 China; ^4^ Department of Orthopedics Guangdong Provincial People's Hospital Guangdong Academy of Medical Sciences Zhongshan 2nd Road 106# Guangzhou 510080 China

**Keywords:** bacterial infections, metal–organic frameworks, photocatalysis, S-scheme heterostructures, wounds healing

## Abstract

Developing new photocatalytic materials is one of the most promising strategies to address bacterial infection during wound healing without bacterial drug resistance, but their poor photocatalytic activity ultimately limits their therapeutic efficacy. In this work, a metal–organic framework‐based S‐scheme heterostructure (Ag_2_S@PB) is prepared to rapidly treat open wounds caused by bacterial infection, which effectively establishes and retains spatially isolated redox centers through an S‐scheme charge‐transfer pathway. Additionally, the formation of an interface electric field and covalent Fe—S bridge bonding in the heterogeneous interface endows the heterojunction with a much stronger charge separation and transfer capability than bare Ag_2_S, which significantly enhances the photocatalytic performance and stability, consequently generating more radical oxygen species. Meanwhile, the introduction of Prussian blue greatly improves the photothermal effect of the Ag_2_S@PB under 808 nm near‐infrared light. Therefore, under illumination for 20 min, the Ag_2_S@PB shows a desirable antibacterial efficiency of 99.92% against *Staphylococcus aureus* and 99.86% against *Escherichia coli*. The in vivo wound repair experiment shows that the Ag_2_S@PB has good tissue repair effects and can expedite wound healing. This work will provide insights for designing highly efficient photoresponsive materials to treat bacterial wound infections.

## Graphical abstract

A Ag_2_S@PB S‐scheme heterojunction, which effectively establishes and preserves spatially isolated redox centers via S‐scheme charge‐transfer paths under near‐infrared light is prepared. Additionally, the Fe–S bonding interface and the internal electric field promote the effective separation of photogenic charge and generate more reactive oxygen species. The enhanced photocatalytic performance combines with the photothermal effect to realize rapid sterilization without antibiotics.

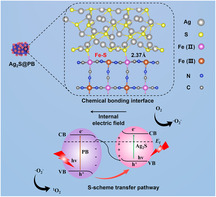

## 1 Introduction

The infection of pathogenic microorganisms induced by bacteria and viruses has been threatening the health and even life safety of people around the world. The COVID‐19 pandemic in 2019, for example, has caused more than 6 million deaths and 628 million infections, in addition to a negative impact on the global economy.^[^ ^1^ ^]^ It should be noted that the leading cause of death is complicated by secondary bacterial infections, particularly drug‐resistant bacterial attacks and infections, instead of the virus itself.^[^ ^2^ ^]^ Currently, the traditional and common treatment for bacterial infections is still antibiotic therapy because of its broad‐spectrum bactericidal properties; however, this promotes the development of bacterial drug resistance and ultimately leads to no effective drugs to use.^[^ ^3^ ^]^ Therefore, it is increasingly important and urgent to explore non‐antibiotic strategies in the struggle against bacterial infections, particularly multi‐drug resistant bacteria.^[^ ^4^ ^]^

In recent years, photothermal therapy (PTT) and photodynamic therapy (PDT) have been extensively used in the treatment of bacterial infections due to their efficient sterilization and lack of bacterial resistance.^[^ ^5^ ^]^ Photothermal and photocatalytic properties, guided by photoresponsive nanomaterials under light irradiation, can quickly eliminate a bacterial infection through the generation of localized hyperthermia and reactive oxygen species (ROS).^[^ ^6^ ^]^ It has been reported that a large number of photoresponsive materials, such as Bi_2_S_3_, MoS_2_, g‐C_3_N_4_, graphene oxide (GO), MXene, and other materials, can be fabricated or modified by diverse methods for use in the PDT, PTT, or synergistic PDT/PTT antibacterial field.^[^ ^7^ ^]^ Among these materials, silver sulfide (Ag_2_S) is considered to be a promising and excellent photocatalytic material that can be activated by near‐infrared (NIR) light irradiation owing to its narrow band gap, high stability, and excellent biocompatibility.^[^ ^8^ ^]^ Consequently, Ag_2_S has been utilized in biomedical fields such as biological imaging and phototherapy.^[^ ^9^ ^]^ However, due to the rapid recombination of photoactivated charges, low efficiency of light absorption, and conversion efficacy, its poor photocatalytic activity has ultimately restricted its application potential.^[^ ^10^ ^]^ Hence, the combination of Ag_2_S and other functional materials with stronger light absorption ability and a suitable bandgap position to construct heterogeneously structured photocatalysts can not only increase the utilization of light but also achieve the separation of photoexcited carriers and promote charge transport to yield more ROS.

Recently, the Prussian blue (PB) metal–organic framework (MOF), a photothermal agent authorized by the US Food and Drug Administration (FDA),^[^ ^11^ ^]^ has attracted attention and been widely applied in various fields including intracellular bioimaging,^[^ ^12^ ^]^ antimicrobial research,^[^ ^13^ ^]^ and tumor diagnosis because of its unique properties,^[^ ^14^ ^]^ such as a simple preparation method, excellent biocompatibility, high biosafety, adjustable functionality, semiconductor behavior, high photothermal conversion property, and NIR‐responsive properties.^[^ ^15^ ^]^ Moreover, the large specific surface area, great chemical stability, and high porosity endow the PB MOF with the ability to adsorb oxygen species effectively.^[^ ^16^ ^]^

**Scheme 1:**
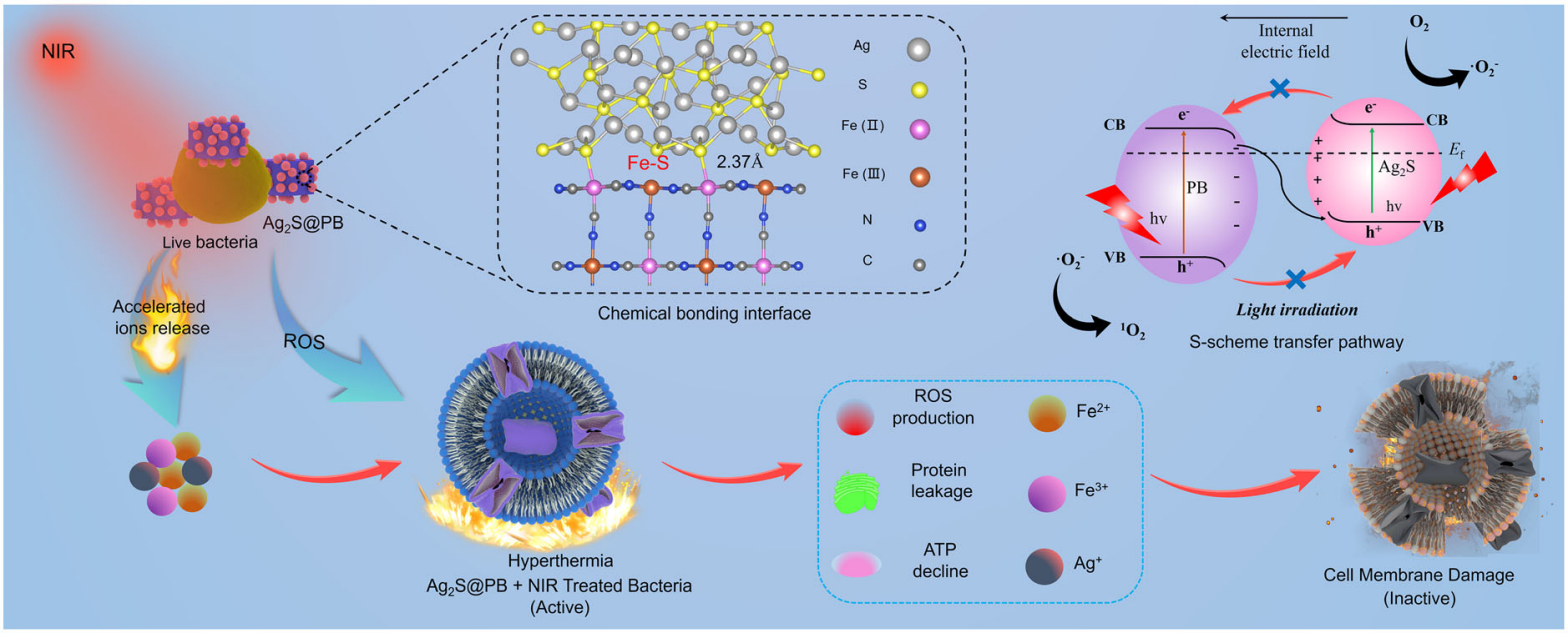
Scheme illustrating the mechanism of Ag_2_S@PB efficiently kills bacteria through 808 nm NIR illumination.

**Figure 1:**
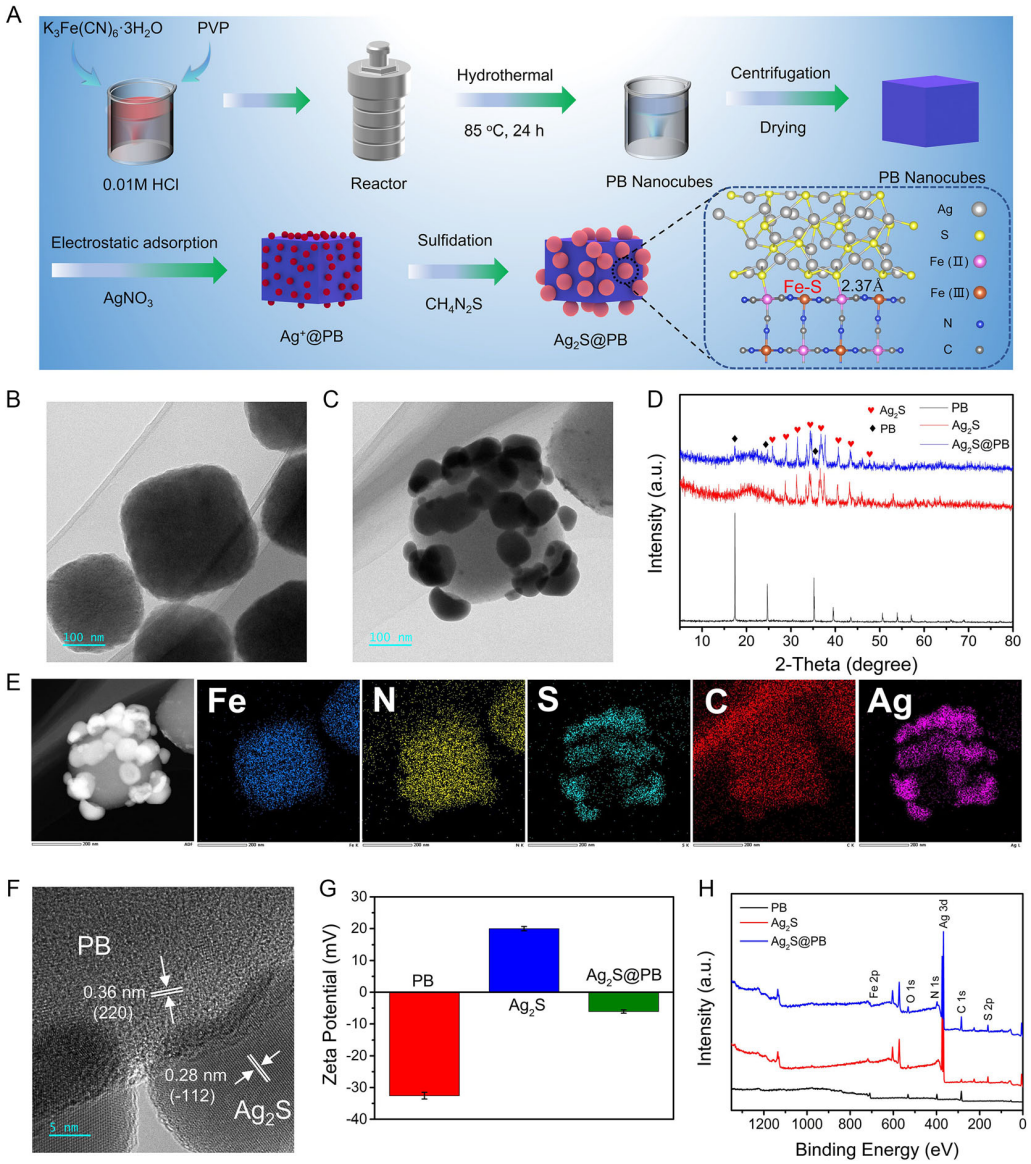
A) Schematic diagram of the fabrication process of Ag_2_S@PB. B,C) TEM of PB MOF (B) and Ag_2_S@PB (C). D) XRD spectra of PB, Ag_2_S, and Ag_2_S@PB. E) TEM EDS mapping and F) HR‐TEM in Ag_2_S@PB. G) Zeta potential of PB, Ag_2_S, and Ag_2_S@PB. H) XPS survey spectra of the materials.

In view of the above background and introduction, we hypothesize whether a cocatalyst of Ag_2_S@PB MOF can be constructed, which showed high photocatalytic and photothermal efficiency, thereby realizing effective antimicrobial behavior at mild temperatures when exposed to 808 nm NIR illumination (**Scheme** **1**). Based on this hypothesis, we designed the Ag_2_S@PB S‐scheme heterojunction consisting of Ag_2_S and PB MOF through electrostatic adsorption and in situ growth method. The preparation procedure of the Ag_2_S@PB is schematically illustrated in **Figure** **1**A. Our results show that the construction of an S‐scheme heterojunction combining n‐type Ag_2_S and n‐type PB favors the production of ROS because of the enhanced separation and migration of photogenic carriers resulting from the differences in Fermi level and band structure. Therefore, Ag_2_S@PB has excellent photocatalytic properties. PB has excellent photothermal performance. The introduction of PB also greatly improved the photothermal performance of Ag_2_S@PB. As shown in Scheme 1, the antibacterial mechanism of Ag_2_S@PB is as follows: when Ag_2_S@PB and bacteria are irradiated by laser at 808 nm, Ag_2_S@PB can produce a large amount of ROS and heat in a short time; at the same time, the increased temperature can accelerate the release of Fe^2+^, Fe^3+^, and Ag^+^. ROS and hyperthermia will peroxidize the lipid membrane of bacteria, damage the cell membrane structure, and increase the permeability of the cell membrane, eventually leading to protein outflow and ATP synthesis is blocked. Second, the release of Fe^2+^, Fe^3+^, and Ag^+^ can cause oxidative stress in bacteria. The combination of ROS, heat, and ions leads to bacterial death.

## 2 Results and Discussion

### 2.1 Synthesis and Characterization of the Materials

The morphology and structure of the as‐prepared materials (PB, Ag_2_S, and Ag_2_S@PB) are displayed in Figure 1B,C,S1, and S2, Supporting Information, respectively. Field‐emission scanning electron microscopy (FE‐SEM) visibly exhibited that PB was a smooth cube and Ag_2_S was a nanosphere. Furthermore, through SEM images of Ag_2_S@PB after 24 h agitation, we can intuitively see that Ag_2_S does not fall off after 24 h of stirring. It can be shown that the Ag_2_S nanospheres firmly anchored PB MOF (Figure S3, Supporting Information). Transmission electron microscopy (TEM) revealed that the average sizes of pristine PB and Ag_2_S@PB were approximately 290 nm and 420 nm, respectively, which were well in line with the measurement results of the dynamic light scattering (DLS) (Figure S4, Supporting Information). DLS exhibited that the average particle sizes of PB and Ag_2_S@PB composite in phosphate buffer saline (PBS) were about 284.0 and 417.6 nm, respectively. The high‐resolution (HR)‐TEM images (Figure S5, Supporting Information) revealed interplanar spacings of 0.24 and 0.19 nm corresponding to the (400) plane of the PB and the (−212) plane of Ag_2_S nanoparticles, respectively, suggesting the in situ formation of Ag_2_S. As shown in Figure 1D, the X‐ray diffraction (XRD) patterns exhibited diffraction peaks at 26.0°, 29.0°, 31.5°, 34.4°, 36.8°, 40.8°, and 43.4°, corresponding to the (−111), (111), (−112), (−121), (121), (031), and (200) crystal faces of Ag_2_S,^[10a]^ respectively, which existed in Ag_2_S@PB, suggesting the successful introduction of Ag_2_S. What was more, the crystal planes of the PB phase including 17.5° (200), 24.3° (220), and 35.2° (400) appeared in Ag_2_S@PB,^[^ ^17^ ^]^ indicating the successful combination of Ag_2_S with PB.

TEM elemental mapping photographs of Ag_2_S@PB (Figure 1E) confirmed that Ag_2_S@PB possessed every element of Ag_2_S (Figure S6, Supporting Information) and PB (Figure S7, Supporting Information), suggesting the existence of these two components. The EDS mapping showed that the elements C, N, and Fe were situated at the core, as well as the Ag and S were uniformly dispersed around the PB MOF, indicating the uniform deposition of Ag_2_S nanoparticles on PB MOF. The crystalline structure of the Ag_2_S@PB is clearly shown in Figure 1F, in which the lattice spacing of 0.36 nm is assigned to the typical face (220) of PB,^[^ ^18^ ^]^ and the lattice spacing of 0.28 nm is assigned to the typical face (−112) of Ag_2_S.^[^ ^19^ ^]^ Also, the zeta potentials of PB, Ag_2_S, and Ag_2_S@PB aqueous solutions were measured to be −32.6, 20.0, and −6.1 mV, respectively (Figure 1G), which confirmed this electrostatic interaction between two components in the synthesis process. The positive Ag^+^ cations were easily absorbed on the surface of negatively charged PB MOF by electrostatic adsorption. Ag_2_S nanoparticles were in situ self‐assembled on the surface of PB MOF at room temperature. The X‐ray photoelectron spectrometer (XPS) survey spectrum (Figure 1H) in PB included the signal peaks of Fe 2p, C 1*s*, N 1*s*, and O 1*s*. The signal peaks of Ag 3d and S 2p are presented in the Ag_2_S@PB spectrum after the combination with Ag_2_S. The above results and analysis demonstrate the successful fabrication of Ag_2_S@PB.

### 2.2 Chemical Bonding Interface in Ag_2_S@PB

**Figure 2:**
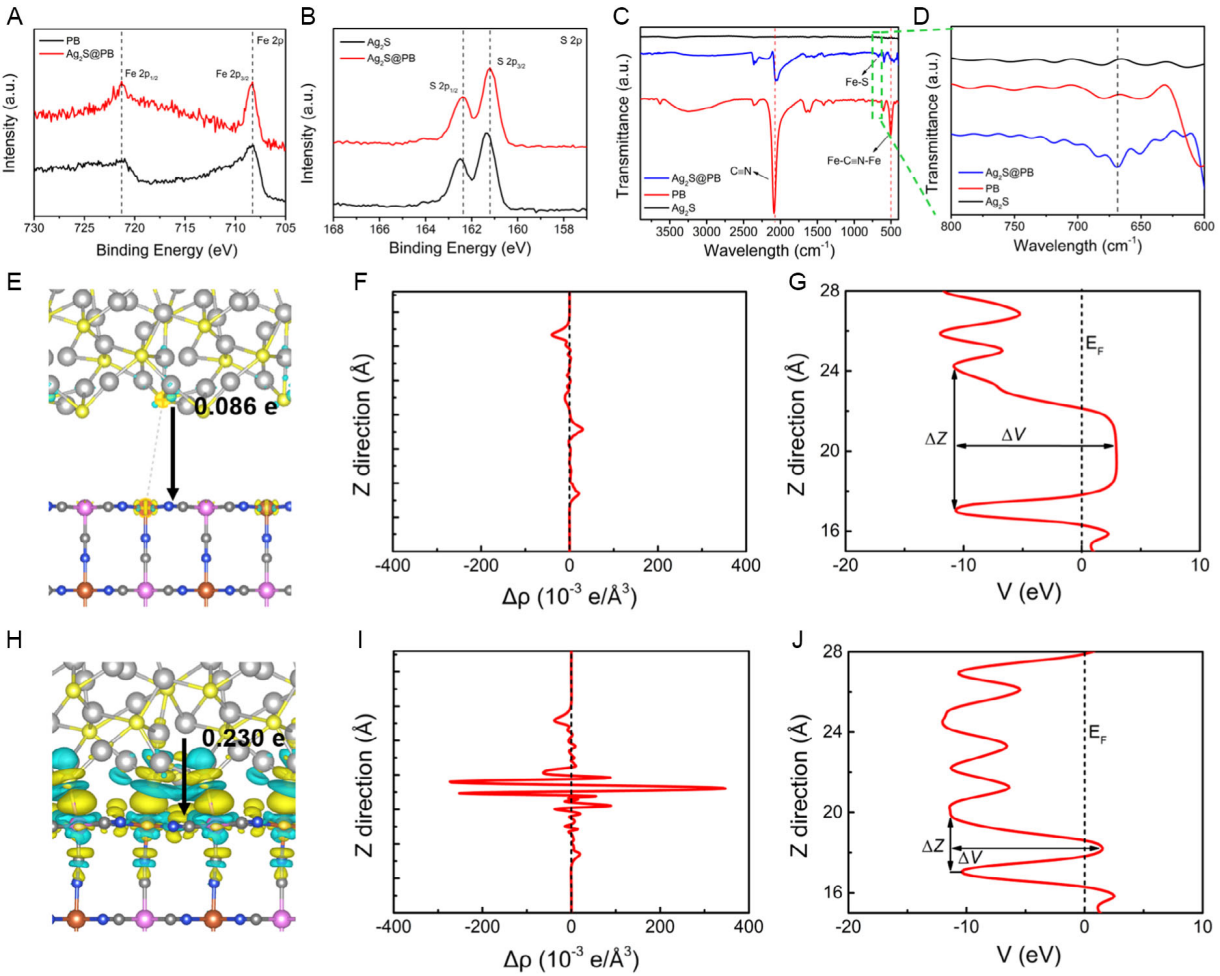
A) Fe 2p XPS spectrometry in PB and Ag_2_S@PB. B) S 2p XPS spectrometry in Ag_2_S and Ag_2_S@PB. C) FTIR spectra of PB, Ag_2_S, and Ag_2_S@PB. D) Partial enlarged FTIR drawing in the wavelength range of 600–800 cm^−1^. E,H) Differential charge density for physically mixing model (E) and chemically bonded model (H). F,I) *<*Δ*ρ*(*z*)*>* averaged by the *xy* face of the layers belonging to various positions on the *z*‐axis. G,J) Electrostatic potential *<V>* averaged by the *xy* face of the layers belonging to various positions on the *z*‐axis.

The high‐resolution spectrum of Fe 2p for Ag_2_S@PB showed two peaks at 708.3 and 721.3 eV, corresponding to Fe 2p_3/2_ and Fe 2p_1/2_ of Fe^2+^,^[^ ^20^ ^]^ separately, while the peaks at 708.2 and 721.0 eV were assigned to the PB (**Figure** **2**A), which exhibited a positive shift in binding energy, indicating an interfacial interaction between PB and Ag_2_S. Meanwhile, as for Ag_2_S@PB, S 2p_3/2_ and S 2p_1/2_ of S 2p at 161.2 and 162.4 eV exhibited a negative shift in binding energy compared with Ag_2_S at 161.3 and 162.5 eV,^[10a]^ suggesting that this could attribute to the formation of Fe—S bonds between PB and Ag_2_S, which were conducive to reducing the recombination of the photo‐inspired carriers (Figure 2B).^[^ ^21^ ^]^ Additionally, compared with PB and Ag_2_S, the existence of new Fe—S bonds in Ag_2_S@PB was further demonstrated by Fourier transform infrared spectrometer (FTIR), with an additional new peak appearing at 668 cm^−1^ corresponding to the stretch of the Fe—S bond (Figure 2C,D).^[^ ^22^ ^]^ Previous research has reported that the work functions of PB and Ag_2_S are 4.85 and 3.90 eV, severally, suggesting a lower Fermi energy level of PB.[^10^, ^16^] When the two phases are in close contact, free electrons spontaneously transfer from Ag_2_S to PB, giving them an identical Fermi level and building an interface electric field at the interface of the two phases. The electronic structure characters of the chemically bonded interface in Ag_2_S@PB were simulated by theoretical computations, while a physical mixing model was established in contrast. In Figure 2E,H, the yellow and blue colors stand for charge excess and charge deficiency, severally. The contour plots confirm that the charge redistribution of Ag_2_S@PB in the chemically bonded interfaces is more intensive and larger.^[^ ^23^ ^]^ Subsequently, Δ*ρ* takes the average value on the *z* = const plane (Figure 2F,I), and the difference signal of the chemical bond interface model increases significantly. The electron transfer number of the models was also quantified through Bader charge analysis. The outcomes show that Ag_2_S transfers 0.230 e electrons to PB via the chemically bonded boundary, whereas the physical hybrid model is merely 0.086 e, confirming that the chemically bonded interface possesses evident priority for the amount of migration charge. Furthermore, in view of the fact that carrier migration is restricted through the interface potential energy barrier, we further probed the zone's electrostatic potential in the heterostructure, as shown in Figure 2G,J. The potential energy barrier in the physical mixing model is Δ*z* ≈ 5.440 Å and Δ*V* = 13.677 eV, while the corresponding Fermi level electrons exist in Δ*z* ≈ 4.326 Å and Δ*V* ≈ 2.920 eV tunnel barriers. At the chemically bonded interface, the potential barrier reduces to Δ*z* ≈ 2.519 Å and Δ*V* = 12.812 eV, while the Fermi barrier reduces to Δ*z* ≈ 0.784 Å and Δ*V* ≈ 1.506 eV. Accordingly, the energy barrier of the chemically bonded interface was smaller, and the charges were easier to transport, suggesting that the chemically bonded interface is conducive to facilitating the transfer of electrons through the heterostructure. Probably, the formation of the Fe—S bond at the interface between the PB and Ag_2_S plays the role of a bridging ligand, indicating that a more explicit interface of Ag_2_S@PB formed between PB and Ag_2_S, potentially with an enhanced charge migration.^[^ ^24^ ^]^ This could certainly boost carrier separation and transmission and be beneficial to the progress of the photocatalytic reaction.

### 2.3 Photocatalytic and Photothermal Performance

**Figure 3:**
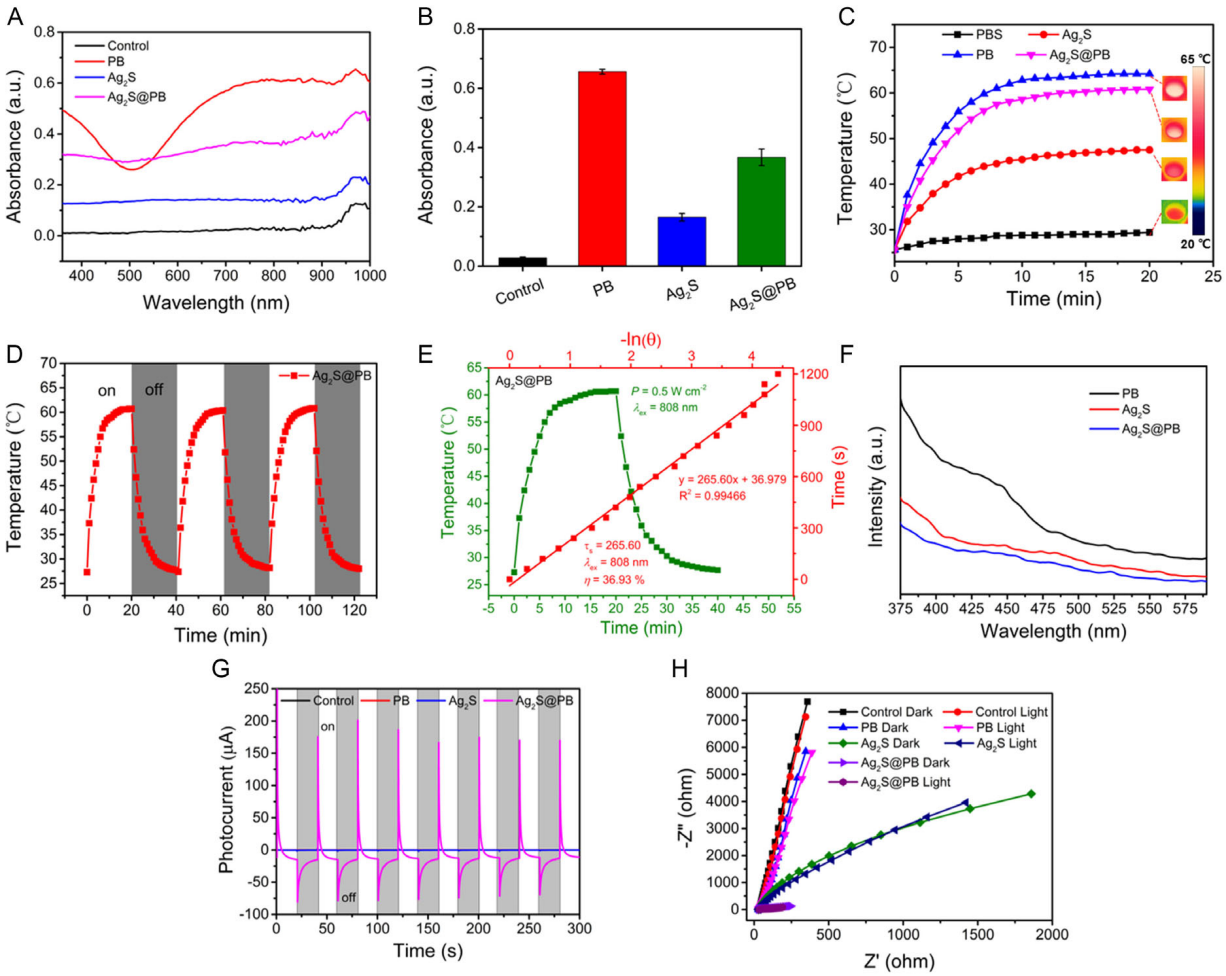
A) Absorption spectrum of material aqueous solution. B) Absorbance of material aqueous solution at 808 nm. C) The heating curve of materials. D) Cycle curve of heating and cooling of Ag_2_S@PB. E) The photothermal conversion efficiency of Ag_2_S@PB. F) PL spectra of materials. G) Transient photocurrent response curves of materials. H) EIS spectra of materials.

The optical performances were analyzed by ultraviolet–visible spectrometry, as depicted in **Figure** **3**A. The outcomes showed that the absorption ability of PB was strongest between 600 and 1000 nm, but the Ag_2_S was weakest. Hence, Ag_2_S@PB possessed a stronger light‐harvesting capacity than Ag_2_S. Because the light absorption capacity of Ag_2_S@PB at 700–1000 nm was significantly strengthened after the introduction of PB MOF (Figure 3A,B). Ag_2_S@PB may exhibit better photothermal properties than Ag_2_S under 808 nm NIR illumination. Therefore, the photothermal performance of the materials was also investigated and is shown in Figure 3C. After all samples were treated using 808 nm NIR illumination (power density: 0.5 W cm^−2^) for 6 min, the temperature of PB and Ag_2_S@PB raised from 25.6 to 58.0 °C and 54.3 °C under NIR laser radiation, respectively. After 20 min of NIR irradiation, the temperature of PB, Ag_2_S, and Ag_2_S@PB rose to 64.2, 47.5, and 60.8 °C, respectively. Comparatively, the temperature of the Ag_2_S group only raised to 42.9 °C, confirming that the introduction of PB remarkably enhanced the photothermal effect of Ag_2_S under 808 nm NIR laser stimulation. The repeatable switching cycle assays showed that Ag_2_S@PB possessed great photothermal stability (Figure 3D). Also, the corresponding photothermal conversion rate (*η*) of Ag_2_S@PB was investigated (Figure 3E). From the relevant time constant (*τ*_s_ = 265.60) and maximum temperature, the photothermal conversion rate of Ag_2_S@PB was counted as 36.93%, further verifying the excellent photostability of Ag_2_S@PB under 808 nm NIR laser exposure. Additionally, the Ag_2_S@PB + light group and Ag_2_S@PB group were shown in Figure S8, Supporting Information, where the amounts of released silver and iron ions in the Ag_2_S@PB + light group are larger than those in the Ag_2_S@PB group. This indicated that the photothermal effect accelerated the release of silver and iron ions in the Ag_2_S@PB.

The photoluminescence (PL) of semiconductors is primarily due to the recombination of photoactivated carriers. Therefore, a fluorescence spectrometer test is generally utilized to investigate the transfer and recombination process of photo‐inspired electrons and holes in materials, and the reduction in fluorescence strength shows that the possibility of electron–hole pair recombination reduction, which suggests that this material possesses a higher photocatalytic activity.^[^ ^25^ ^]^ The PL analysis in Figure 3F shows that the fluorescent light emission at around 450 nm in Ag_2_S@PB was significantly weaker than Ag_2_S and PB. The lower fluorescence intensity indicated a smaller recombination rate of photogenic carriers for Ag_2_S@PB. This outcome might imply the fast migration of photo‐inspired electrons from Ag_2_S to PB at the cladding interface, suppressing the recombination of photoactivated carriers. Accordingly, the photocarrier separation effect of the Ag_2_S@PB nanocomposite was enhanced.^[^ ^26^ ^]^ Moreover, the photocurrent and electrochemical impedance spectrum (EIS) were adopted to confirm this outcome. The photocurrent was examined to estimate the carrier transmission capacity. As shown in Figure 3G and S9, Supporting Information, the photocurrent curves of PB, Ag_2_S, and Ag_2_S@PB showed great repeatability under 808 nm NIR light excitation, in which the photocurrent intensity of Ag_2_S@PB was significantly larger than PB and Ag_2_S, suggesting a larger photocarrier separation efficiency of Ag_2_S@PB.^[^ ^27^ ^]^ Therefore, Ag_2_S@PB showed the best photocatalytic activity. The EIS array was employed to probe the impedance of the samples.^[^ ^28^ ^]^ As observed in Figure 3H, Ag_2_S@PB exhibited the lowest arc, suggesting that Ag_2_S@PB possessed the largest photocarrier separation rate and the lowest migration resistance. In addition, lower impedances in the light groups were shown compared with the darkness groups. The above outcomes and discussion validated that the photocatalytic properties of Ag_2_S@PB were dramatically strengthened through the combination of Ag_2_S and PB, thereby contributing to the yield of ROS.

**Figure 4:**
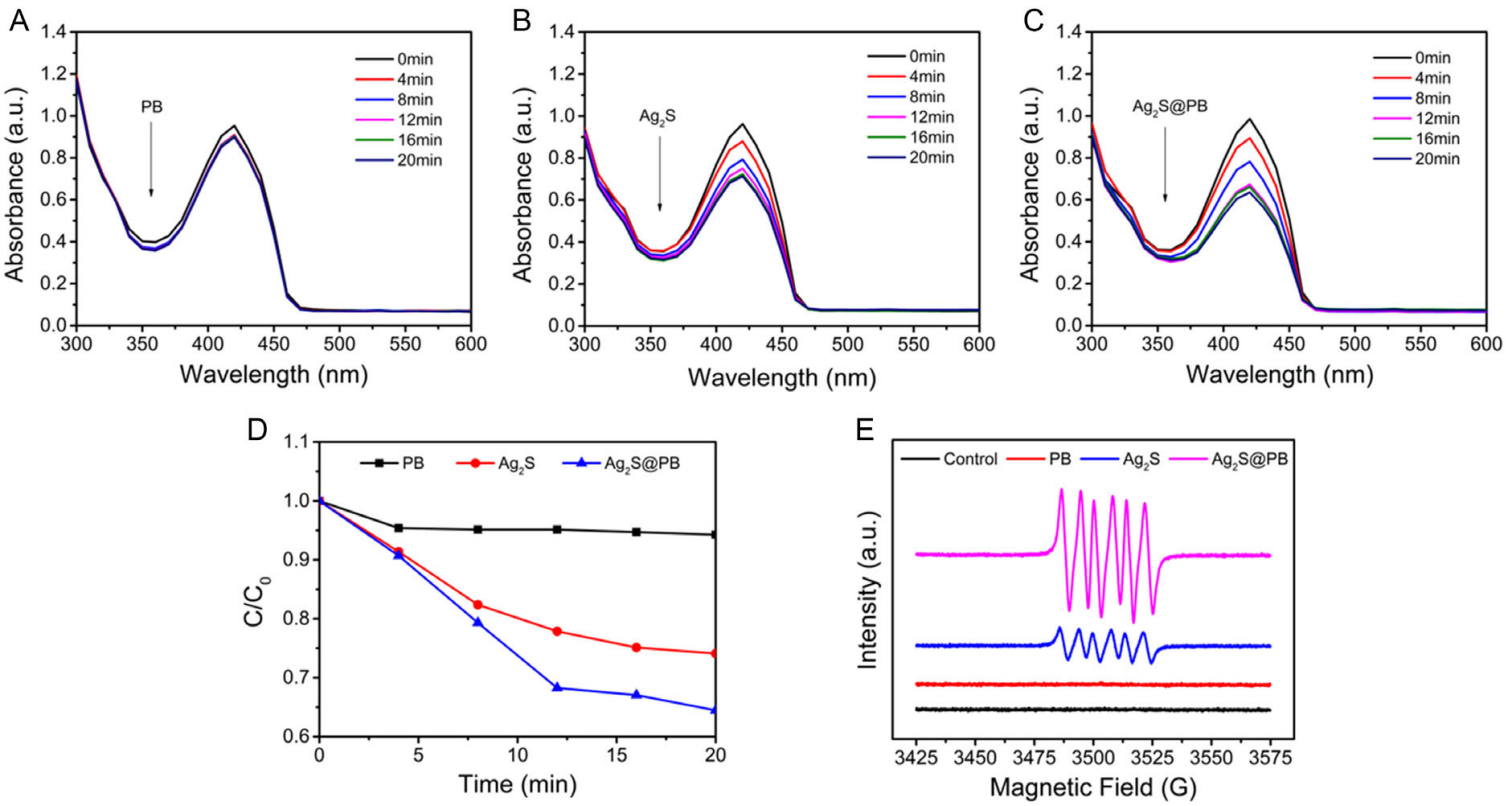
A–C) DPBF degradation was used to monitor ^1^O_2_ of PB (A), Ag_2_S (B), and Ag_2_S@PB (C) under light treatment. D) The comparison curves of DPBF degradation of PB, Ag_2_S, and Ag_2_S@PB. E) The ESR spectra of materials.

As a singlet oxygen (^1^O_2_) collector, 1,3‐diphenylisobenzofuran (DPBF) was adopted to monitor the ^1^O_2_ yields of the materials using 808 nm NIR light illumination. The DPBF absorption peak strength at 425 nm was descended while specificity combined with ^1^O_2_.^[^ ^29^ ^]^ As shown in **Figure** **4**A, the DPBF absorption peak strength in PB remained almost unchanged with the increase in illumination time, indicating that PB hardly generates ^1^O_2_ under light exposure. Comparatively, the absorption strength of the Ag_2_S@PB and Ag_2_S groups at 425 nm declined stage by stage, and Ag_2_S@PB descended faster than Ag_2_S, suggesting that the incorporation of PB remarkably strengthened the photocatalytic activity of Ag_2_S (Figure 4B,C). Furthermore, the corresponding contrast curve visibly indicated that Ag_2_S@PB showed greater ^1^O_2_ production than Ag_2_S (Figure 4D and S10, Supporting Information). To further explore ROS types, electron spin resonance (ESR) was conducted to monitor the superoxide anion (·O_2_^−^) yield of materials (PB, Ag_2_S, and Ag_2_S@PB) under NIR light stimulation.^[^ ^30^ ^]^ As shown in Figure 4E, only PB could not produce ·O_2_^−^ under 808 nm NIR laser irradiation, whereas Ag_2_S and Ag_2_S@PB could produce ·O_2_^−^ under NIR light exposure. The ·O_2_^−^ yield of Ag_2_S@PB increased significantly after the combination with PB, confirming that the addition of PB MOF clearly strengthened the photocatalytic activity of Ag_2_S.

### 2.4 Photocatalytic Mechanism

**Figure 5:**
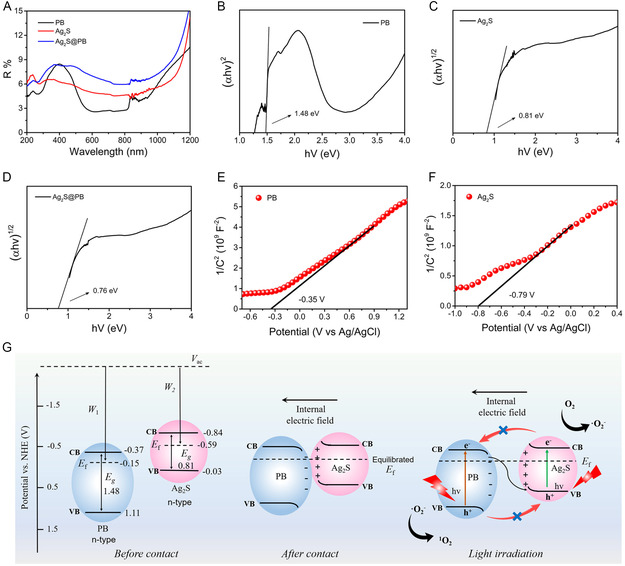
A) UV–vis diffuse reflectance spectrometer of materials. B–D) The bandgap of PB (B), Ag_2_S (C), and Ag_2_S@PB (D). E,F) Mott–Schottky plots of PB (E) and Ag_2_S (F). G) Schematic illustration of S‐scheme charge‐transfer mechanism and photocatalytic mechanism of the ROS generation under 808 nm NIR illumination.

To estimate the generation and transmission of photoexcited carriers, the bandgap energy of the materials was measured. **Figure** **5**A displays the UV–vis diffuse reflectance spectrometer of PB, Ag_2_S, and the Ag_2_S@PB composite. According to the UV–vis diffuse reflectance spectrometer and the Kubelka–Munk function versus energy gap, the bandgap of PB, Ag_2_S, and Ag_2_S@PB were calculated as 1.48, 0.81, and 0.76 eV, respectively (Figure 5B–D), indicating that the energy gap of Ag_2_S@PB was dramatically redshifted compared with Ag_2_S. This is conducive to the production of more photogenerated charges through 808 nm light stimulation.^[^ ^31^ ^]^ Previous literature reported that the valence band (VB) edge potential and conduction band (CB) edge potential of Ag_2_S were −0.03 and −0.84 eV vs normal hydrogen electrode (NHE).^[10a]^ Similarly, the VB edge potential and CB edge potential of PB were −0.37 and 1.11 eV versus NHE had also been reported.^[^ ^16^ ^]^ Further information was acquired using Mott–Schottky tests, such as the Fermi energy level (*E*_f_). As observed in Figure 5E,F, the slopes of the tangent lines on PB and Ag_2_S are positive, indicating that they are both n‐type semiconductors.^[^ ^32^ ^]^ From the intersection of the tangent line as well as *y* = 0 plot, the specific flat band potentials (*V*_fb_, vs Ag/AgCl) of PB and Ag_2_S are about −0.35 and −0.79 V, corresponding to −0.15 and −0.59 V versus NHE, respectively.^[^ ^33^ ^]^ In general, the value of *V*_fb_ is close to the *E*_f_,^[^ ^34^ ^]^ so the electronic band structures of PB and Ag_2_S are elucidated in Figure 5G. Compared with PB, Ag_2_S has a higher band position and Fermi energy level. When PB is in close contact with Ag_2_S, free electrons in Ag_2_S spontaneously migrate to PB through the interface until their Fermi energy levels align.^[^ ^35^ ^]^ During equilibrium, electrons accumulate at the PB interface, and the electron density at the Ag_2_S interface decreases at the same time, resulting in downward bending of the energy band on PB and upward bending of the energy band on Ag_2_S.^[^ ^36^ ^]^ Therefore, a built‐in electric field is constructed on the boundary of the two phases, with the electric field direction pointing from Ag_2_S to PB. Under 808 nm NIR light excitation, electrons in PB and Ag_2_S are activated from the VB to the CB, respectively. The inner electric field drives the photo‐inspired electrons of PB to consume the photo‐inspired holes of Ag_2_S, leaving photo‐inspired holes and electrons in the VB of PB and the CB of Ag_2_S, severally. Obviously, this charge‐transfer process is an S‐scheme mechanism, which not only effectively retains the strong reduction capacity of the CB photoexcited electrons in Ag_2_S but also effectively retains the strong oxidation capacity of the VB holes in PB, thereby exerting a strong driving force for ROS generation.^[^ ^37^ ^]^

Also, due to the higher CB potential (−0.84 V vs NHE) of Ag_2_S compared to the standard potential (−0.33 V vs NHE) of O_2_/O_2_^−^, photoexcited electrons will react with O_2_ molecules to produce ·O_2_^−^,^[^ ^38^ ^]^ which can then further react with photoexcited holes to produce ^1^O_2_.^[^ ^39^ ^]^ In the Ag_2_S@PB, only ^1^O_2_ was produced due to the production of hydroxyl radicals (·OH) at the higher VB position of the photocatalyst than the VB position of the H_2_O/OH pair (+1.99 V vs NHE). Comparatively, the VB position of the synthetic material was only 1.11 V vs NHE, or obviously lower than the VB position of H_2_O/·OH pair. Hence, no ·OH can be produced.^[^ ^40^ ^]^

### 2.5 Antibacterial Activity and Disinfection Underlying Mechanism

**Figure 6:**
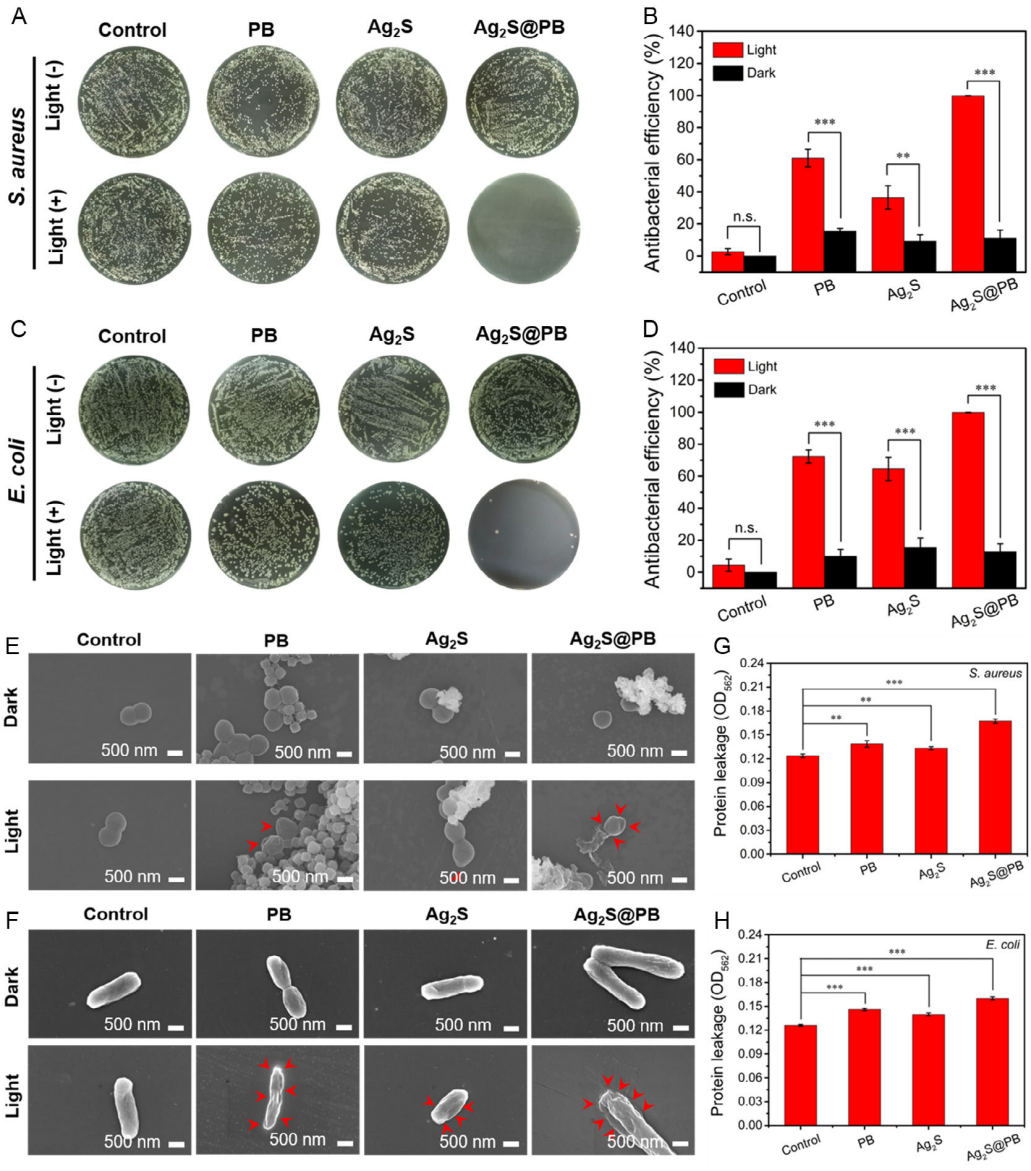
A,C) Antimicrobial outcomes against *S. aureus* (A) and *E. coli* (C). B,D) Bacterial‐killing efficacies of materials toward *S. aureus* (B) and *E. coli* (D). E,F) SEM morphologies photographs against *S. aureus* (E) and *E. coli* (F). G,H) Protein leakage toward *S. aureus* (G) and *E. coli* (H) under light stimulation. *p < 0.05, **p < 0.01, ***p < 0.001.

Gram‐positive *Staphylococcus aureus* and gram‐negative *Escherichia coli* were exploited as representative pathogens to probe the bacterial‐killing capacity of the materials. First, we evaluated the influence of different ratios of PB and Ag_2_S on the antibacterial effect in Ag_2_S@PB. As shown in Figure S11, Supporting Information, when the proportion of PB and Ag_2_S is 15%, the antibacterial effect is the best, up to 99.92%. Therefore, Ag_2_S@PB with this ratio is selected as the final material. The bacterial‐killing performance of the as‐synthesized samples (PB, Ag_2_S, and Ag_2_S@PB) in vitro was evaluated by the plate counting method (**Figure** **6**A,C). In Figure 6B,D, the bactericidal activity in relevant light groups was obviously strengthened compared to the dark groups. In the darkness, the bactericidal effects of PB, Ag_2_S, as well as Ag_2_S@PB were calculated as 15.41%, 9.26%, and 11.02% against *S. aureus* and 9.99%, 15.66%, and 12.87% against *E. coli*, respectively. The low antimicrobial efficacies of PB, Ag_2_S, and Ag_2_S@PB in the dark came from the partial silver ions or iron ions release. By comparison, the antimicrobial ratios of PB, Ag_2_S, and Ag_2_S@PB were 61.01%, 36.44%, and 99.92% against *S. aureus* and 72.39%, 64.60%, and 99.86% against *E. coli* after 808 nm NIR illumination for 20 min. The bacteria‐killing experimental data indicated that the Ag_2_S@PB group possessed the best antibacterial performance following 808 nm NIR light excitation. To intuitively observe the germicidal properties of the materials, we observed the live and dead states of the bacteria under different conditions through live and dead staining experiments. As shown in Figure S12, Supporting Information, the groups without 808 nm NIR light irradiation of all the materials and the Ag_2_S and PB light (+) groups showed partial red fluorescence. In contrast, the Ag_2_S@PB light (+) group exhibited full red fluorescence, compared to the green fluorescence of the control group. To investigate the influence of hyperthermia and ROS on antibacterial performance, the antibacterial experiment was carried out by dividing into the following four groups: control, Ag_2_S@PB + light (19 °C), Ag_2_S@PB + water‐bath (55 °C), and Ag_2_S@PB + light (55 °C). The corresponding antibacterial efficiencies of Ag_2_S@PB + light (19 °C), Ag_2_S@PB + water‐bath (55 °C), and Ag_2_S@PB + light (55 °C) were 31.58%, 62.51%, and 99.91% shown in Figure S13, Supporting Information, respectively, which suggested hyperthermia alone has a higher bactericidal efficiency than ROS alone, and the antibacterial mode alone cannot achieve complete and effective killing of bacteria. Furthermore, a single model including PTT or PDT could not completely and effectively eliminate the bacterial strains. The combination of PTT with PDT showed better bacterial‐killing capacity against *S. aureus* and *E. coli* than PTT or PDT alone. In addition, the Ag_2_S@PB exhibited a cyclical bacteria‐killing effect of over 99% after three circulations of antibacterial measurements (Figure S14B,D, Supporting Information), and the relevant spread plate photographs are displayed in Figure S14A,C, Supporting Information. Based on the above results, Ag_2_S@PB displayed great circulatory germicidal ability and reproducibility.

To visualize the bactericidal properties of the materials, FE‐SEM was applied to observe the surface morphologies of bacteria after various treatments (Figure 6E,F). The surface of the bacteria in the material and control groups was smooth and intact, and the bacterial membrane was not obviously damaged in the darkness, suggesting that the materials had no obvious antibacterial properties under dark conditions. By comparison, the bacterial cell membranes of the PB and Ag_2_S were damaged and creased to varying degrees after exposure to 808 nm illumination for 20 min. What is more, the germ membranes in the Ag_2_S@PB suffered a more serious shrinkage and rupture (signaled with red arrows) because of the synergistic action of moderate ROS and temperature generated by the Ag_2_S@PB under light. Because the destruction of bacterial cell membranes is generally associated with severe protein leakage, this synergistic action on *S. aureus* and *E. coli* could be further demonstrated by protein leakage experiments. In comparison with PB and Ag_2_S (Figure 6G,H), the Ag_2_S@PB with the highest antibacterial effect showed the most leakage of proteins after illumination, which was also consistent with the sterilization outcomes and bacterial morphologies.

**Figure 7:**
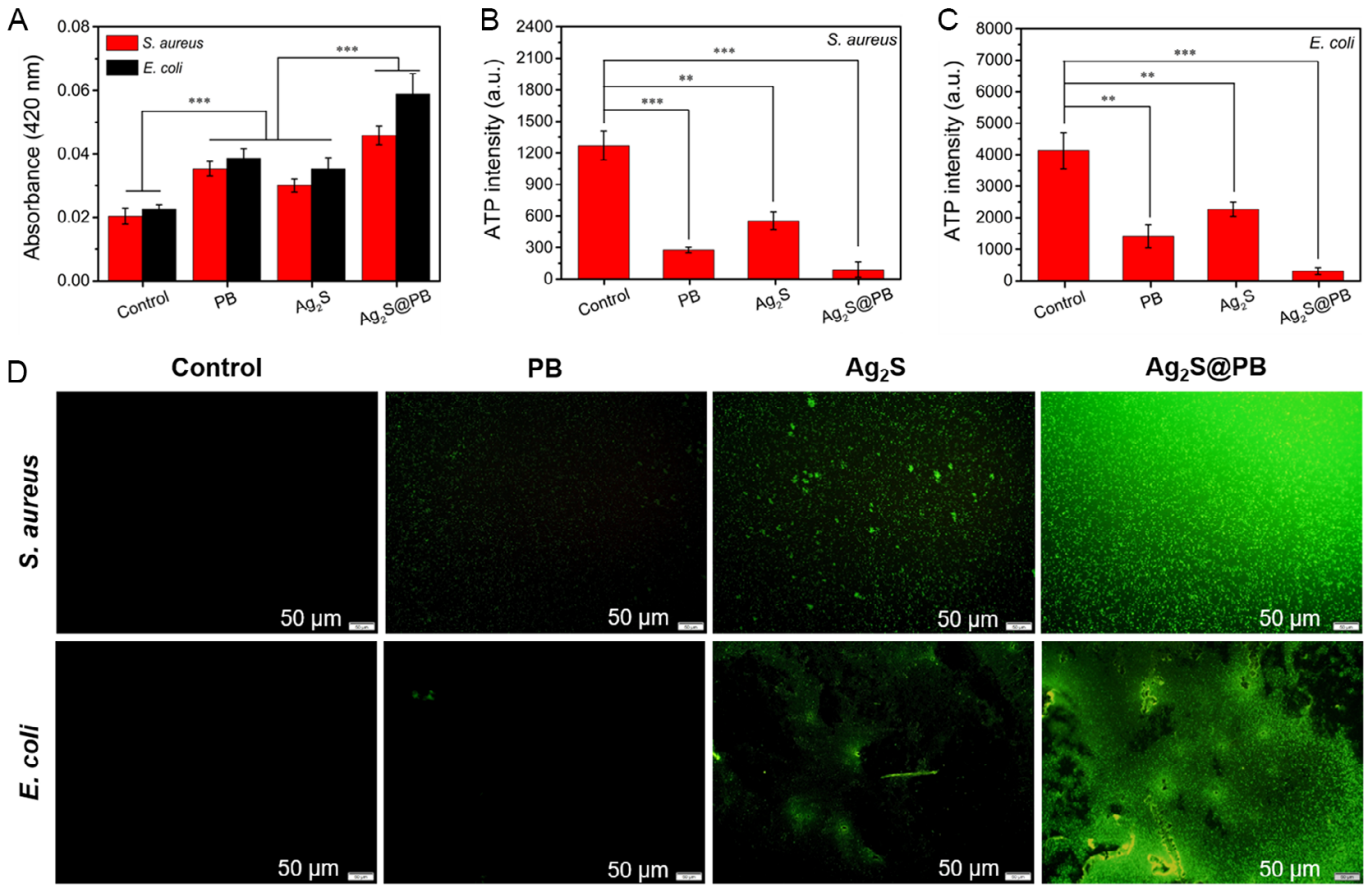
A) ONPG permeability assay of bacterial membrane. B,C) ATP deletion level of *S. aureus* (B) and *E. coli* (C). D) Intracellular ROS was examined with DCFH‐DA. *p < 0.05, **p < 0.01, ***p < 0.001.

Furthermore, the permeability of the bacterial membrane was analyzed to further investigate the main antibacterial mechanism through the *ortho*‐nitrophenyl‐β‐galactoside (ONPG) hydrolysis method. Generally, a higher OD_420nm_ value in ONPG hydrolysis experiments means greater membrane permeability.^[^ ^41^ ^]^ Under light, the bacterial membrane permeability of the material groups (PB, Ag_2_S, and Ag_2_S@PB) increased markedly compared to the control, and the Ag_2_S@PB group showed a larger OD_420nm_ value than the PB and Ag_2_S groups (**Figure** **7**A). This result indicates that the synergistic effect of moderate active oxygen and high temperature can effectively elevate the permeability of the membrane toward *S. aureus* and *E. coli*, so that iron and silver ions can more easily penetrate the bacterial cell membrane. ATP, an important source of energy, participates in many metastasis processes in bacterial intracellular reactions.^[^ ^42^ ^]^ ATP levels in bacteria will decline after bacterial necrosis or apoptosis.^[^ ^43^ ^]^ As shown in Figure 7B,C, the ATP levels in the apoptosis of bacteria incubated with different samples under treatment through 808 nm NIR light. The ATP level in the Ag_2_S@PB exhibited the lowest level toward *S. aureus* and *E. coli* compared with the control, PB, and Ag_2_S groups, thus suggesting the excellent bacteria‐killing activity of this kind of material. In addition, a 2′,7′‐dichlorofluorescein diacetate (DCFH‐DA) was exploited to detect intracellular ROS within the bacteria. As depicted in Figure 7D, the strongest green fluorescent light was shown inside bacteria of Ag_2_S@PB group after coculturing *S. aureus* or *E. coli* with the material under light, indicating that the Ag_2_S@PB had the largest level of intracellular ROS among the samples. Namely, an increase in intracellular ROS can induce severe bacterial damage and physiological disorders, thereby accelerating the death of bacteria.^[^ ^44^ ^]^ From the above results and discussion, the Ag_2_S@PB could quickly and efficiently eliminate bacteria under 808 nm NIR illumination because of the synergy of mild hyperpyrexia, proper ROS, and the released silver and iron ions.

### 2.6 In Vitro Cytocompatibility Evaluation

**Figure 8:**
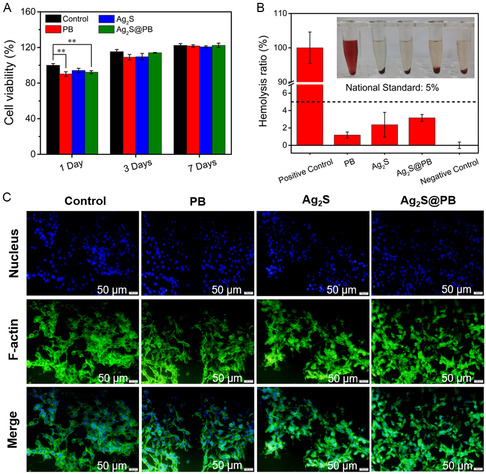
A) Cell viability at 1, 3, and 7 days of cultivation. B) Hemolysis test of materials. C) Fluorescence images of cells cultivated with samples (scale bar = 50 μm). *p < 0.05, **p < 0.01, ***p < 0.001.

The biocompatibility of materials for 1, 3, and 7 days was estimated using the 3‐(4,5‐dimethylthiazol‐2‐yl)‐2,5‐diphenyltetrazolium bromide (MTT) assay. As observed in **Figure** **8**A, the Ag_2_S (94.20%) showed larger cell viability than PB (90.27%) and Ag_2_S@PB (92.28%) after 1 day of incubation in comparison with the control. The cell viability in the PB group displayed a slightly lower survival rate due to the excessive release of iron ions. However, the survival rates of the PB, Ag_2_S, and Ag_2_S@PB groups were all above 95% after 3 and 7 days of coincubation with the extension of incubation time. The above experimental results showed the excellent biocompatibility of PB, Ag_2_S, and Ag_2_S@PB. Additionally, the hemolysis test was exploited to assess the hemolytic performances of materials. As shown in Figure 8B, the hemolysis rates of PB, Ag_2_S, and Ag_2_S@PB were all lower than the internationally recognized standard (5%), suggesting that the Ag_2_S@PB possessed great blood compatibility.^[^ ^45^ ^]^ Moreover, a cell fluorescence assay was employed to intuitively estimate the cytotoxicity of the materials, as shown in Figure 8C. From the cell fluorescence images, it could be visualized that the NIH‐3T3 cells in both the control group and the material groups were relatively complete in morphology, and an abundant cellular extension state with a polygonal morphology suggested that the cells grew well. The abovementioned results and discussion further confirmed that the materials were not cytotoxic and possessed excellent biocompatibility. Figure S15A,B, Supporting Information, shows the behavior of silver and iron ions cumulatively released from Ag_2_S@PB within 14 days in vitro, and the final concentrations of silver and iron ions were 0.048 and 1.355 mg L^−1^, respectively. The cumulative release of silver ions is very low, and there is no obvious biological toxicity. In addition, Fe is not only a vital biological element for physiological activities but also the released Fe ions can facilitate cell differentiation by secreting collagen.^[^ ^46^ ^]^ Therefore, the material possessed excellent cytocompatibility.

### 2.7 In Vivo Antimicrobial Assay and Wound Healing

**Figure 9:**
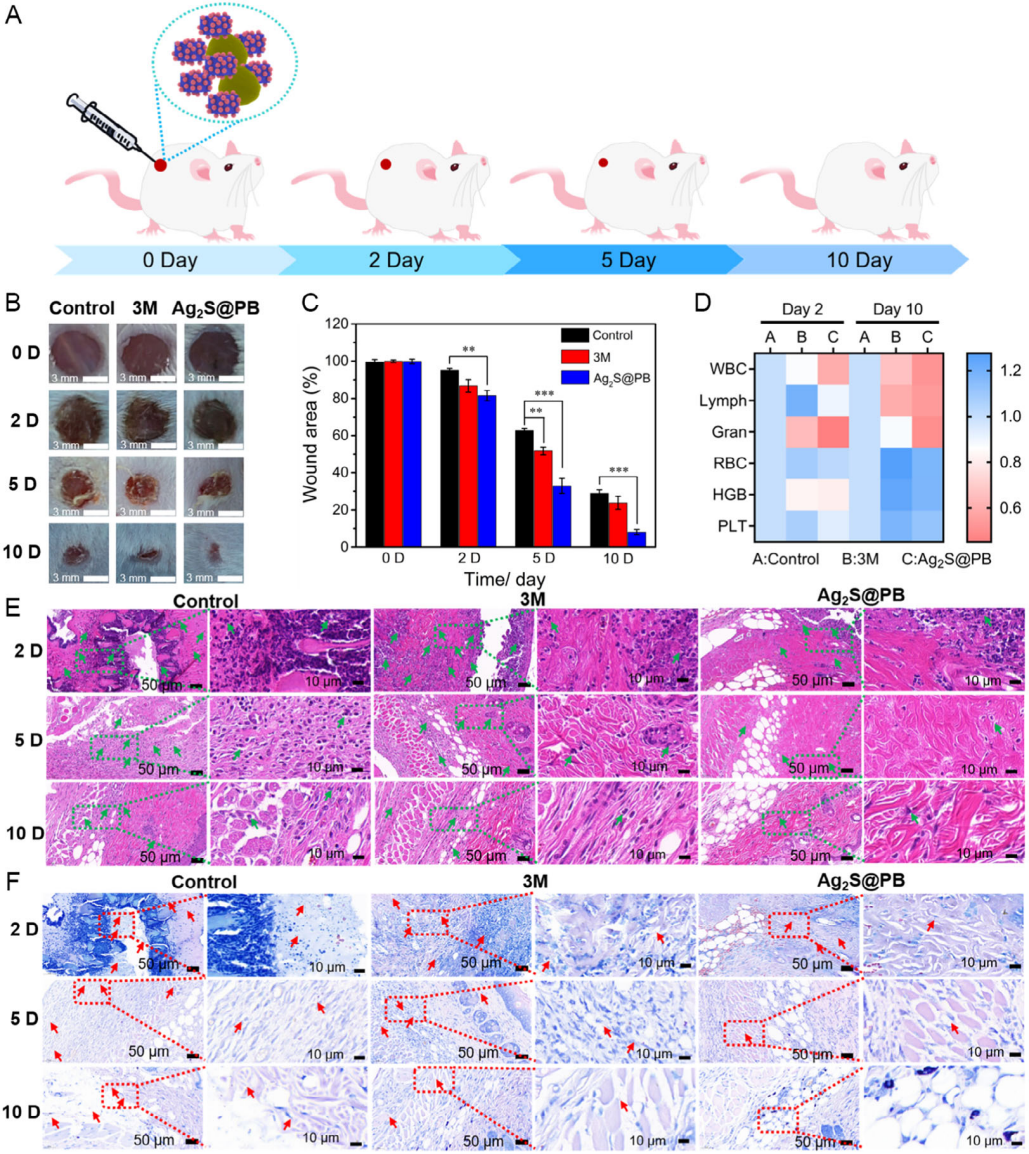
A) Schematic diagram of the antibacterial effect of Ag_2_S@PB in a wound infection model. B) Representative photographs of mice wound at 0, 2, 5, and 10 days for control, 3M, and Ag_2_S@PB groups. C) Corresponding wound healing process at 0, 2, 5, and 10 days. D) Blood routine at 2 and 10 days. E) The H&E staining images and F) Giemsa staining images in infected wounds at 2, 5, and 10 days (scale bars = 50 μm). *p < 0.05, **p < 0.01, ***p < 0.001.

The wound infection model of mice was built to assess the antibacterial performance in vivo. The therapeutic effect of Ag_2_S@PB on *S. aureus* wound infection in mice is illustrated in **Figure** **9**A. First, we used the plate‐coating method to detect the changes in the number of colonies on the back wounds of different groups of mice before and after treatment. The experimental results showed that bacterial infection on the back wound of Ag_2_S@PB NIR group treatment was almost completely cleared, 3M dressing (3M) treatment did not have a rapid antibacterial effect, so the bacterial clearance rate of back wounds in the 3M group was very low after 20 min of treatment. In the control group, the bacterial clearance rate of the back wound was the lowest (Figure S16, Supporting Information). These results suggest that Ag_2_S@PB can quickly and effectively remove bacterial infection from back wounds in mice. The diagrams of wound healing on different days exhibited that the wound in the Ag_2_S@PB group nearly closed through 10 days of therapy, whereas the wounds of the control and 3M groups still possessed apparent defects (Figure 9B). Correspondingly, the wound area chart displayed that the wounds of Ag_2_S@PB were always smaller than the control and 3M at different time points (Figure 9C). The blood routine and blood chemistry at 2 and 10 days evaluated the bacterial infection. As displayed in Figure 9D, the values including the white blood cells, granulocytes (gran), and lymphocytes (lymph) of the control and 3M were visibly larger than those in the Ag_2_S@PB, which indicated the Ag_2_S@PB group possessed the lowest intensity of inflammatory response in vivo after surgery. Beyond that, the red blood cells (RBCs), platelets, and hemoglobin of these three groups had similar results, suggesting that Ag_2_S@PB had no in vivo cell cytotoxicity. Hence, Ag_2_S@PB can kill bacteria under light and does not cause bacterial infection.

**Figure 10:**
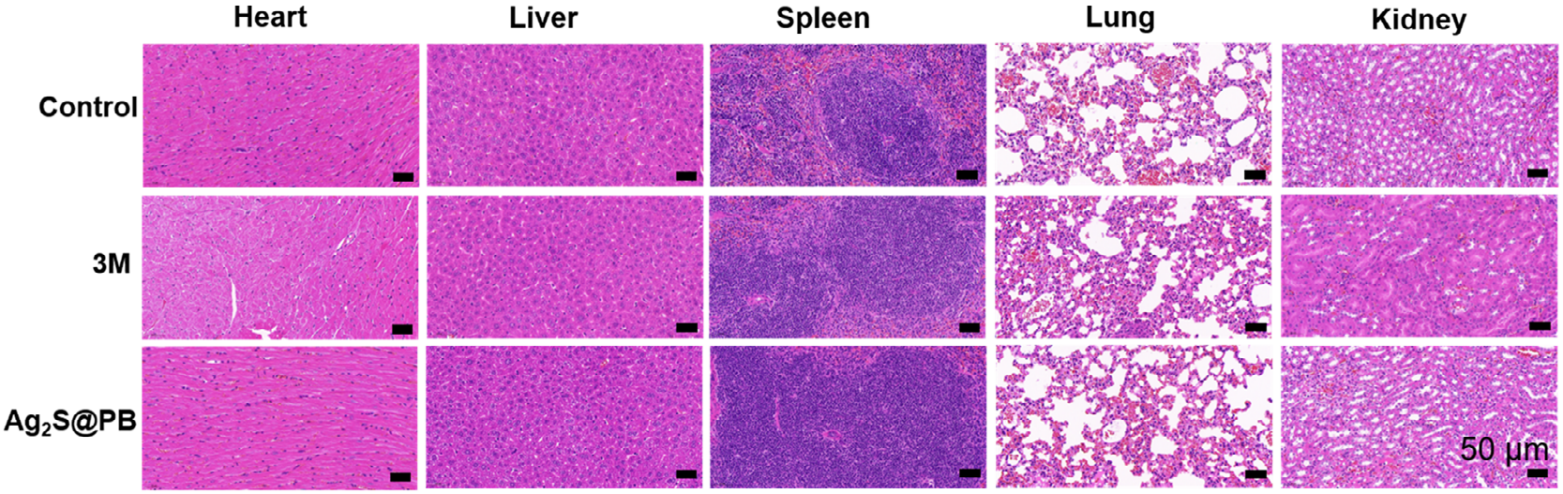
H&E staining images of the main organs at 10 days (scale bars = 50 μm).

As shown in Figure 9E, the inflammatory cells of Ag_2_S@PB were obviously lower than the control and 3M at 2, 5, and 10 days, indicating that the inflammatory response on the wound tissue was lower in the material group. Giemsa staining was employed to observe the amounts of bacteria adhering to the wound site. As shown in Figure 9F, after treatment of 2, 5, and 10 days, it can be seen from the wound tissue slices that the germs of the Ag_2_S@PB were obviously less than the control and 3M, suggesting that the level of bacterial infection at the wound site in the sample groups was lower. In addition, Masson staining at 2 and 10 days is shown in Figure S17, Supporting Information. More collagen fibers were observed in the Ag_2_S@PB under illumination, indicating the best tissue regeneration and wound‐healing situation. In contrast, rough and irregular collagen was observed in the control and 3M because of tissue inflammation and obvious bacterial residue in the infected tissues. Therefore, these assay outcomes indicated that Ag_2_S@PB possesses excellent bactericidal and tissue‐healing capacity at the infected site. As displayed in **Figure** **10**, the H&E staining was conducted including the heart, lung, kidney, liver, and spleen in the mouse to assess the toxicology of Ag_2_S@PB in vivo. The main organs in the Ag_2_S@PB were normal and had no visible organ damage, suggesting that the Ag_2_S@PB possessed good biosecurity in vivo.

## 3 Conclusion

A multifunctional S‐scheme Ag_2_S@PB heterojunction photocatalyst was fabricated for the first time and applied for rapid bacteria‐killing. Due to the strong photothermal effect and enhanced photocatalysis capacity of Ag_2_S@PB, this nanomaterial exhibits excellent bactericidal activity under 808 nm light irradiation. The mechanism probe of bacteria‐killing showed that the powerful bacteria‐killing ability of this system could be attributed to disrupting membrane integrity, generating ROS, and reducing ATP, as well as destroying bacterial metabolism. The rapid and highly efficient antimicrobial effects, together with excellent biocompatibility, make Ag_2_S@PB very promising as a NIR light‐excited disinfectant.

## 4 Experimental Section

4.1

4.1.1

##### Fabrication of PB

The PB nanoparticles were synthesized by a simple hydrothermal synthesis. Briefly, 0.4 g of K_3_[Fe(CN)_6_] and 10 g of polypyrrolidone were added into 160 mL of hydrochloric acid (HCl, 0.1 m) with stirring for 1 h. Afterward, the hybrid was positioned in an oven and heated at 85 °C for 24 h. The as‐formed products were centrifuged (11 000 rpm, 20 min) and rinsed several times using water and ethanol. The resulting PB nanoparticles were dried for 6 h at 50 °C and collected for further use.

##### Fabrication of Ag_2_S and Ag_2_S@PB

PB nanoparticles were weighed according to the different mass ratios (5%, 15%, and 25%) of PB to Ag_2_S, and then added in 10 mL of water for ultrasonic dispersion. 10 mL of AgNO_3_ water solution (0.3 m) was pipetted into the above homogeneous solution with magnetically stirring for 1 h. Subsequently, 10 mL of water solution dissolving 0.15 g of thiourea was dropwise dripped to the above hybrid to induce the reaction. The reaction solution was magnetically stirred at normal temperature for 5 h to fully react. After the reaction, the product was centrifuged by a centrifuge, rinsed with absolute ethanol and water several times, positioned in a −4 °C freezer for 24 h, dried in the lyophilizer for 12 h, and finally ground into a powder sample. For comparison, the synthesis method of Ag_2_S was the same as the above method, except that PB was not added.

##### Characterization of Samples

X‐ray photoelectron spectrometer analysis was measured using the instrument (XPS, ESCALAB 250Xi). The morphologies and microstructures were investigated using FE‐SEM (sigma 500) and TEM (Tecnai G20). The absorption spectrum and absorbance of material aqueous solution at 808 nm were analyzed on the multifunctional enzyme marking instrument (SpectraMax i3, Molecular Devices). The surface potential and size distribution were detected using DLS instrument (Malvern). The crystalline structure was characterized through XRD (D8A25) with step sizes of 0.01°. PL spectra were characterized using fluorescence spectroscopy (PL, LS‐55). The functional groups were obtained through the Fourier transform infrared analysis (FTIR, NICOLET IS10). The diffuse reflectance spectrometer was tested using a UV–vis–NIR spectroscopy (UV‐3600, Shimadzu). Ion release was detected with an inductively coupled plasma optical emission analysis (ICP, PQ9000).

##### The Photoluminescence

The sample (Ag_2_S, PB, Ag_2_S@PB) was prepared with deionized water at a concentration of 200 ppm, and the emission spectrum of the material was obtained by the fluorescence spectrometer (LS‐55, Perkin Elmer, USA).

##### Theoretical Calculations Method

The Vienna Ab initio Simulation Package (VASP) was conducted with density functional theory (DFT) within the generalized gradient approximation (GGA) through the Perdew–Burke–Ernzerhof (PBE) functional. The projected augmented wave potentials were exploited to depict the ionic cores while applying a face wave basis set with a kinetic energy cutoff of 400 eV to consider the valence electrons. The spin‐polarization effect was also considered. The geometry optimizations were carried out in the case of the force convergence lower than 0.05 eV Å^−1^. The van der Waals interactions were depicted using the DFT‐D3 empirical correction analysis. Monkhorst–Pack k‐points of 4 × 4 × 1 were exploited for all the calculations. Atoms at the bottom are fixed in all the computations. After geometry optimization, the charge density mappings, Bader charge, averaged by the *xy* face of the layers belonging to various positions on the *z*‐axis, and the electrostatic potential <*V*> averaged by the *xy* face of the layers belonging to various positions on the *z*‐axis were calculated.

##### Photothermal Property Evaluations

The real‐time temperatures of PB, Ag_2_S, and Ag_2_S@PB (200 μg mL^−1^) were separately recorded using FLIR E50 (Estonia) under 808 nm NIR illumination (20 min, 0.5 W cm^−2^) every minute together with photothermal photographs were taken. The temperature variation of Ag_2_S@PB was also examined within three‐cycle curves. The photothermal conversion rate (*η*) of Ag_2_S@PB is counted according to the following equation:^[^ ^47^ ^]^

(1) $$\eta =\;[hS\times \;(T_{\text{max}}-T_{0})-Q]/[I\times \;(1-10^{-A})]$$

where the parameters of *h*, *S*, *Q*, *A*, *T*_max_, *T*_0_, *I*, and *η* are the heating conversion modulus, the heated surface region, the heating absorption energy of the well plate, the absorbance of Ag_2_S@PB at 808 nm, the highest temperature, the surrounding temperature, and the 808 nm NIR light power, severally. The time constant (*τ*_s_) is counted in accordance with the following formula in time of the cooling period

(2) $$t=-\tau_{s}\times \text{ ln}\theta =\text{ ln}(T-T_{0})/(T_{\text{max}}-T_{0})$$

At this point, if the temperature in this system is steady and the input heat is equivalent to the output heat, the formula is applied as follows:

(3) $$hS=m_{s}\times C_{s}/\tau_{s}$$

where *m*_s_ and *C*_s_ represent the mass as well as the specific heat capacity of water, severally.

##### Photoelectrochemical Tests

On the electrochemical workstation (CHI660E), the photoelectrochemical performances of the material are detected with Pt electrode, Ag/AgCl electrode, and the material as the counter electrode, the reference electrode, and the working electrode, severally. The photocurrent curves and EIS tests were analyzed using 808 nm NIR light (0.5 W cm^−2^) as the light source, as well as Na_2_SO_4_ water solution (0.5 m) as the electrolyte.

##### Photodynamic Property Evaluations

To detect ^1^O_2_ generated by the samples (PB, Ag_2_S, and Ag_2_S@PB) with a concentration of 200 ppm under 808 nm NIR stimulation, DPBF was exploited. When reacting with ^1^O_2_, the fluorescence intensity of DPBF at 420 nm decreased. A 100 μL amount of sample aqueous solution (400 μg mL^−1^) and DMSO solution containing DPBF (100 μL, 50 μg mL^−1^) were added into 96‐well plate, and then the mixtures were treated by 808 nm NIR irradiation for 20 min; the fluorescence intensity change of DPBF was detected every 4 min. To monitor ·O_2_^−^, the ESR (JES‐FA200) was carried out and the 5,5‐dimethyl‐1‐pyrrolin‐N‐oxide was adopted for the ·O_2_^−^ detect agent. 40 μL of Ag_2_S@PB solution (1 mg mL^−1^) and 160 μL of detect agent were treated by NIR light illumination.

##### Antimicrobial Properties

The germicidal activity of PB, Ag_2_S, and Ag_2_S@PB was estimated by the spread plate assay. The materials and equipment in the assay were disinfected by irradiating them with ultraviolet light for 0.5 h in advance. Next, 20 μL of the sample (2 mg mL^−1^) was equably mixed with bacterial diluent (180 μL, 1 × 10^7^ CFU mL^−1^) into a 96‐well plate. The mixture was treated in the dark or using 808 nm NIR light (0.5 W cm^−2^) for 20 min. Afterward, the bacteria fluid was diluted 100 times, and then 20 μL of the above bacterial fluid was evenly smeared. The agar plate was cultured in a 37 °C oven for 24 h. The bacteria‐killing rate was calculated in compliance with the count result of bacteria colonies. To evaluate the influence of hyperthermia or ROS alone on the antibacterial performance of Ag_2_S@PB, the experiments were divided into four groups: control, Ag_2_S@PB + light (19 °C), Ag_2_S@PB + water‐bath (55 °C), and Ag_2_S@PB + light (55 °C). Notably, the temperature of the Ag_2_S@PB + water‐bath (55 °C) and Ag_2_S@PB + light (55 °C) increased for 7 min and kept at 55 °C for 13 min, while the Ag_2_S@PB + light (19 °C) group maintained at 19 °C warming and cooling balance in an ice‐water bath.

##### Bacterial Morphology

After the bacteria‐killing assay was completed, the medium of the orifice plate was discarded and washed for three times. Next, 2.5% glutaraldehyde was added to immobilize bacteria and washed with PBS, then gradient ethanol (30%, 50%, 70%, 90%, and 100%) was successively used and dehydrated. After the orifice plate was dried, the morphologies of bacteria were exhibited using SEM.

##### Evaluation of Protein Leakage and Membrane Permeability

The bacterial protein leakage was quantitatively examined by the method of bichinchonic acid (BCA) protein analysis. Simply put, after the antibacterial assay was completed, the 150 μL of bacterial diluent and 150 μL of PBS were mixed, and then rinsed by centrifuge (5 min, 6000 rpm). Finally, 25 μL of liquid supernatant was pipetted into 200 μL of BCA reagent. The corresponding OD value at OD_562 nm_ was examined using the microplate analyzer.

The permeability of the cell membrane was determined using the nitrobenzo‐β‐galactoside (ONPG) test. After the germicidal assay, the ONPG detection kit was employed to monitor the absorbance of suspension at 420 nm by the microplate analyzer.

##### ATP Activity Test

The ATP activity on *S. aureus* and *E. coli* was detected using ATP enhancers. First, the bacterial suspension was washed three times in a centrifuge at 4 °C with PBS, and diluted to 10^8^ CFU mL^−1^ using PBS. The material (400 ppm) was mixed with the above bacterial diluent in a ratio of 1:1, and the supernatant was centrifuged and discarded after treatment with 808 nm NIR illumination for 20 min. The ATP lysis buffer was then pipetted into the precipitate, which was dispersed in the ultrasonic cell disruptor for 5 min. Afterward, the liquid supernatant was obtained by centrifuge and mixed with ATP working solution in a 96‐well plate at a ratio of 1:1. Finally, the fluorescence intensity was determined by the microplate analyzer.

##### Intracellular ROS Determination

Intracellular ROS kit (cat# S0033; Beyotime) was used in this assy. In short, 10^7^ CFU mL^−1^ bacteria and DCFH‐DA diluter (10 mm) were incubated in darkness in a 37 °C shaker for 30 min, and then materials were added for another 4 h of coculture. After that, the bacteria were left standing for 1 h, then discarded the medium and washed off the excess dye with PBS for several times. After natural drying, fluorescence pictures were obtained by an inverted fluorescence microscope.

##### In Vitro Cytocompatibility Evaluation

The NIH‐3T3 fibroblasts were incubated in Dulbecco's modified Eagle medium at 37 °C. The cells and samples were incubated after 1, 3, and 7 days, severally. Then, the cell culture fluid was replaced with MTT (0.5 mg mL^−1^) suspension and cultured in an incubator after 4 h. Subsequently, the MTT suspension was discarded and displaced by DMSO solution with a shake for 20 min. Finally, the OD values at 570 nm as well as 490 nm of the supernatant were determined.

The cell fluorescence assay was carried out to intuitively observe cell morphology. The fibroblasts and samples were incubated in the cell incubator for 1 day. The fibroblasts were washed and fixed with 4% formaldehyde after discarding the cell medium. The fibroblasts were dyed with fluorescein isothiocyanate (FITC) in the dark and washed. Then, the cells were dyed by 4,6‐diamino‐2‐phenyl indole (DAPI) and then rinsed. The cell morphology of the sample surface was observed on an inverted fluorescence microscope.

##### Hemolytic Rate Test

The hemolysis test of the samples was carried out with fresh rats’ blood. The blood was centrifuged at 3000 rpm for 15 min at 4 °C, and the supernatant was removed. The RBCs were collected and rinsed with PBS three times, and then dispersed in PBS for storage. Subsequently, we mixed 1 mL of a PBS solution of samples (400 ppm) with 1 mL of a 10% RBC dispersion. The mixed solution was then cultured at 37 °C for 4 h, and the absorbance of the supernatant at 570 nm was measured with a microplate reader after centrifugation at 3000 rpm for 15 min. The positive control is water, and the negative control is PBS. The hemolytic rates (RHR%) of samples were calculated according to the following formula:

(4) $$\text{RHR }\left(\%\right)\;=\;\left(A_{\text{sample}}-\;A_{\text{PBS}}\right)/\left(A_{\text{water}}-\;A_{\text{PBS}}\right)$$

##### Animal Experiments In Vivo

BALB/c male mice were obtained in the Animal Hospital of Huazhong Agricultural University. The animal assay protocol was authorized by the Animal Research Committee of Tongji Medical College, Huazhong University of Science and Technology, Wuhan. The animal assays were carried out in compliance with the Regulations on Animal Management of the Ministry of Health of the People's Republic of China and Guidelines for the Care and Use of Laboratory Animals in China. The 30 mice were randomly categorized: control, 3M (3M medical dressing for wound dressing of mice), and Ag_2_S@PB group, with 10 mice in each group. After anesthesia, a wound with a 6 mm diameter was cut on each mouse's back, and 10 μL of *S. aureus* (10^8^ CFU mL^−1^) solution was dropped to create an infection model. After 2 h, 10 μL of Ag_2_S@PB sample (0.4 mg mL^−1^) was dropped into the back wound of mice in Ag_2_S@PB group for light treatment (808 nm NIR, 20 min), 10 μL of normal saline was dropped into the back wound of mice in control group, and then wrapped with ordinary dressing. 10 μL doses of normal saline were dropped into the back wound of the 3M group of mice, and then 3M was applied to bind the wound. The wounds in mice were photographed at 2, 5, and 10 days and taken for Masson staining, Giemsa staining, as well as H&E staining, respectively. In addition, the heart, liver, spleen, lung, and kidney of mice were treated with the H&E staining to evaluate the internal biocompatibility of samples for 10 days.

##### Statistical Analysis

The *p*‐values were determined by unpaired *t*‐test, one‐way analysis of variance (ANOVA) with Dunnett's multiple comparisons test, or two‐way ANOVA with Dunnett's multiple comparisons test or with Sidak's multiple comparisons test. The data were presented as mean values + standard deviations (SD). The value of SD indicated error bar. When *p < 0.05, it was considered statistically significant. The *n* number of biologically independent samples in each group was at least three. Exact *n* values are marked in the images.

## Conflict of Interest

The authors declare no conflict of interest.

## Supporting information

Supplementary Material

## Data Availability

The data that support the findings of this study are available from the corresponding author upon reasonable request.
