# Supplementary material for: The Enhanced Photocatalytic Sterilization of a Metal–Organic‐Framework‐Based S‐Scheme Heterostructure of Ag2S@PB for Rapid Healing of Bacteria‐Infected Open Wounds
Source: Small Sci. 2023 Nov 20;3(12):2300114. doi: 10.1002/smsc.202300114 (PMC11935958; doi:10.1002/smsc.202300114)
Supplement: Supplementary file 1 — Supplementary Material [file SMSC-3-2300114-s001.pdf]

Supporting Information

The Enhanced Photocatalytic Sterilization of a Metal–Organic-Framework-Based S-Scheme Heterostructure of Ag_2S@PB for Rapid Healing of Bacteria-Infected Open Wounds

*Zhenxing Yang, Cuihong Chen, Chaofeng Wang, Yufeng Zheng, Shuilin Wu, Yu Zhang, Xiangmei Liu**

Mr. Z. Yang, Miss. C. Chen, Prof. X. Liu
Biomedical Materials Engineering Research Center, Hubei Key Laboratory of Polymer Materials, Ministry-of-Education Key Laboratory for the Green Preparation and Application of Functional Materials, School of Materials Science & Engineering, Hubei University, Wuhan 430062, China
E-mail: liuxiangmei1978@163.com (X. Liu)

Mr. C. W, Prof. X. Liu
School of Health Science & Biomedical Engineering, Hebei University of Technology, Xiping Avenue 5340#, Tianjin, 300401, China
E-mail: liuxiangmei1978@163.com (X. Liu)

Prof. Y. Zheng, Prof. S. Wu
School of Materials Science & Engineering, Peking University, Yiheyuan Road 5#, Beijing, 100871, China

Prof. Y. Zhang
Department of Orthopedics, Guangdong Provincial People's Hospital, Guangdong Academy of Medical Sciences, Zhongshan 2nd Road 106#, Guangzhou, 510080, China

*Corresponding author

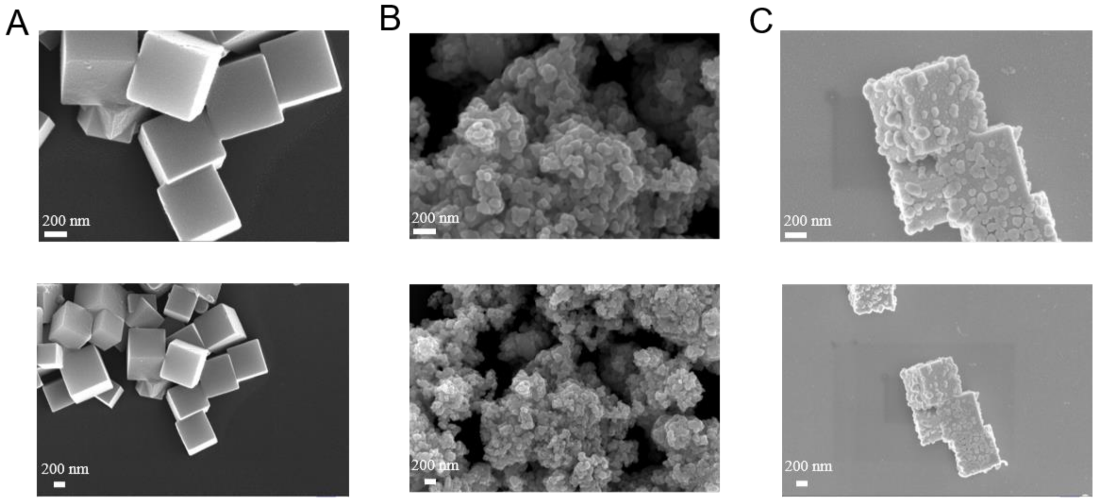

Figure S1. FR-SEM of (A) PB, (B) Ag_2S, and (C) Ag_2S@PB.

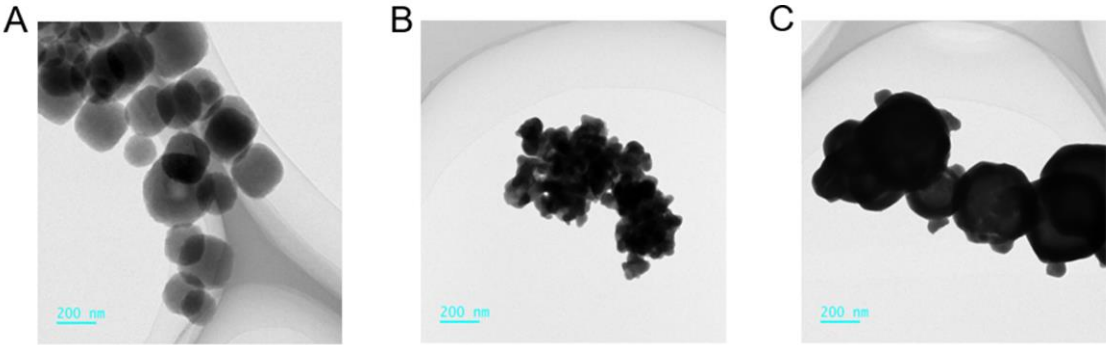

Figure S2. TEM of PB (A), Ag_2S (B), and Ag_2S@PB (C).

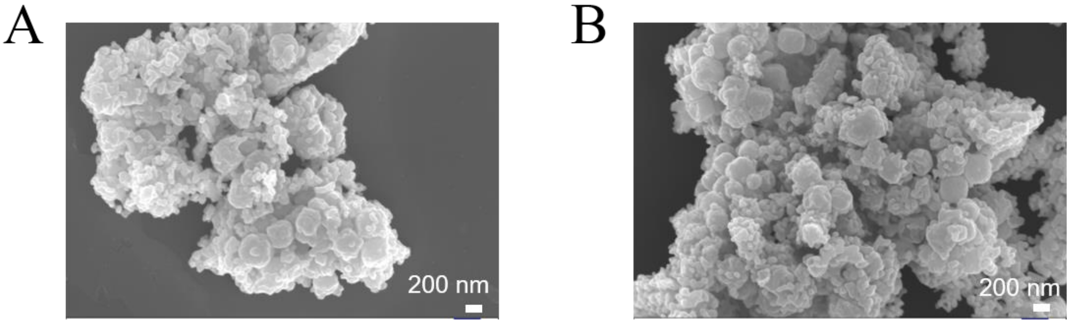

Figure S3. (A) The picture of Ag_2S@PB before stirring for 24 h in PBS solution. (B) The picture of Ag_2S@PB after stirring for 24 h in PBS solution.

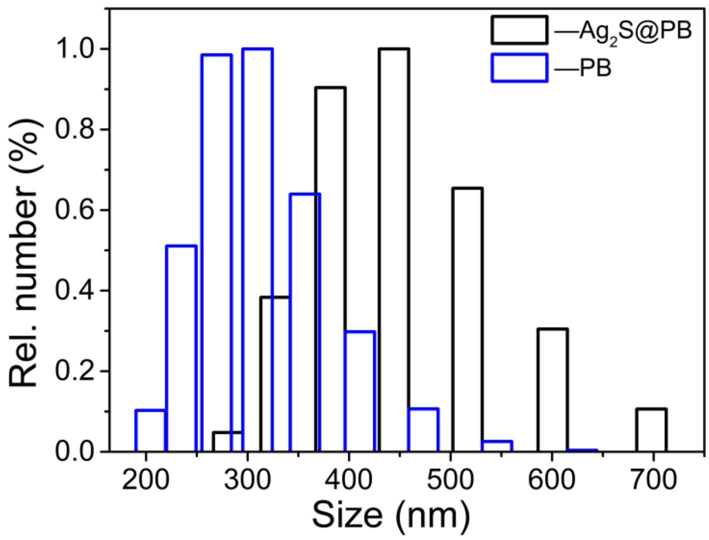

Figure S4. The size distribution of PB and Ag_2S@PB.

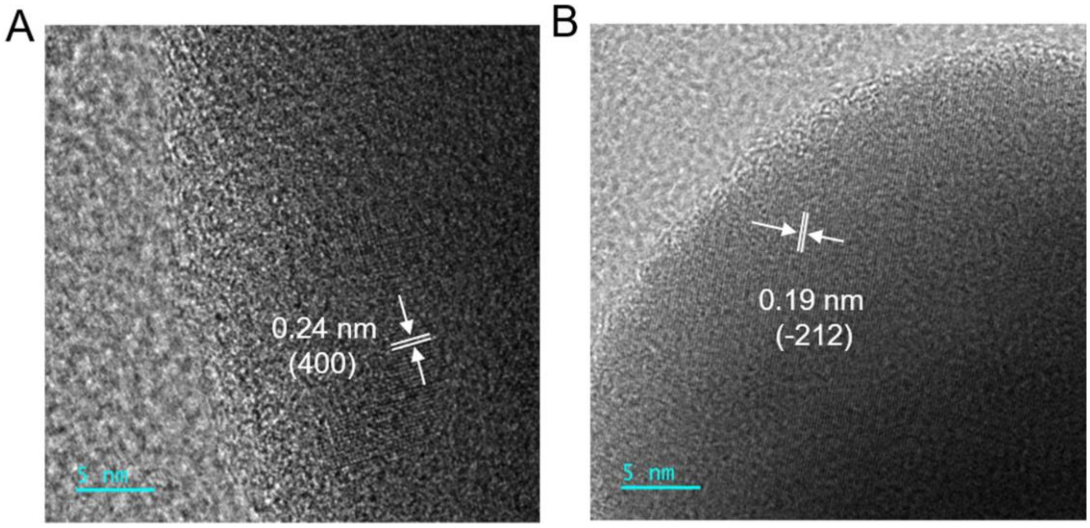

Figure S5. HRTEM images of (A) PB and (B) Ag_2S.

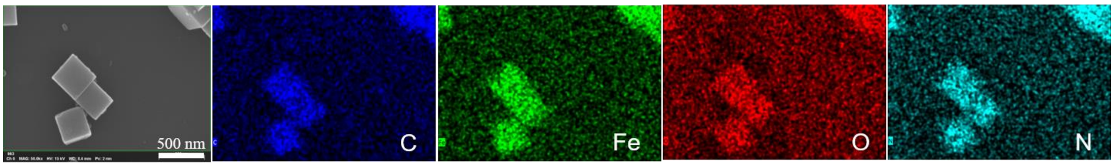

Figure S6. FR-SEM Mapping of Ag_2S.

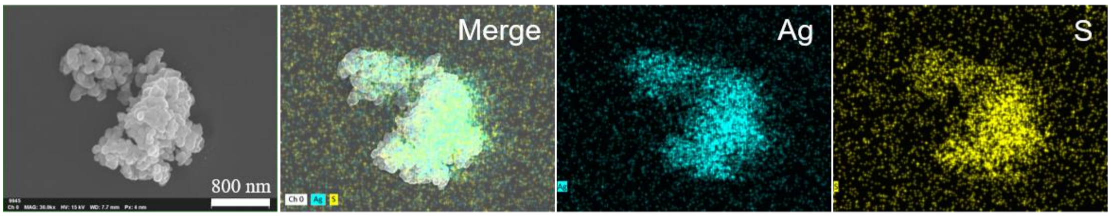

Figure S7. FR-SEM Mapping of PB.

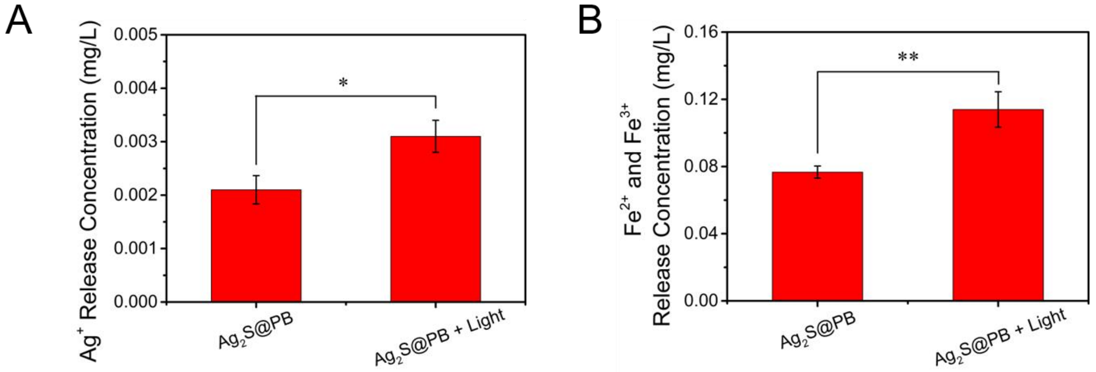

Figure S8. The release of (A) silver and (B) iron ions in the Ag_2S@PB with NIR light irradiation or without light irradiation.

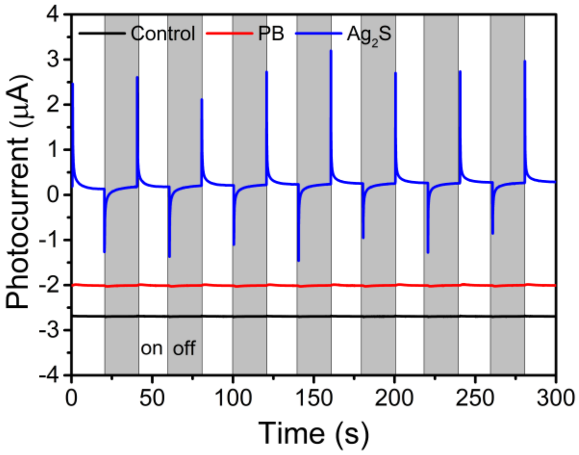

Figure S9. Transient photocurrent response curves of materials.

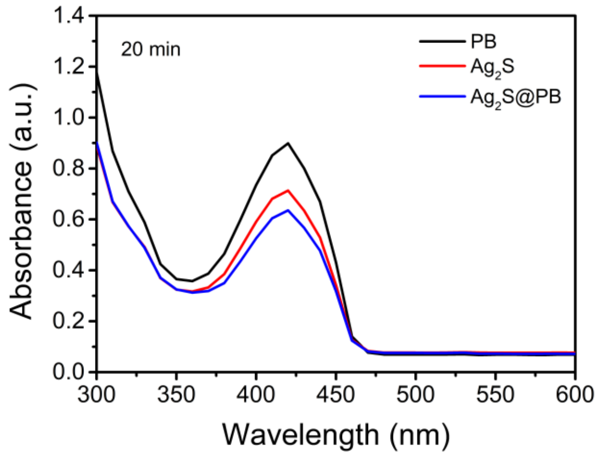

Figure S10. DPBF degradation was used to monitor the $^1O_2$ of PB, Ag_2S, and Ag_2S@PB at the 20th minute of light treatment.

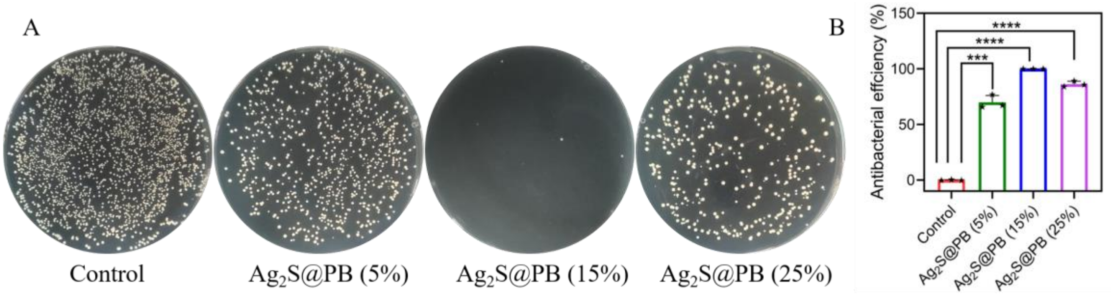

Figure S11. (A) Images of different ratios of PB and Ag_2S on the antibacterial effect in Ag_2S@PB against *S. aureus* under 808 nm near-infrared light. (B) The calculated germicidal efficiencies towards *S. aureus* in accordance with the spread plate counts. The error bars stand for means ± SD, n = 3. ns > 0.05, *p < 0.05, **p < 0.01, ***p < 0.001, ****p < 0.0001.

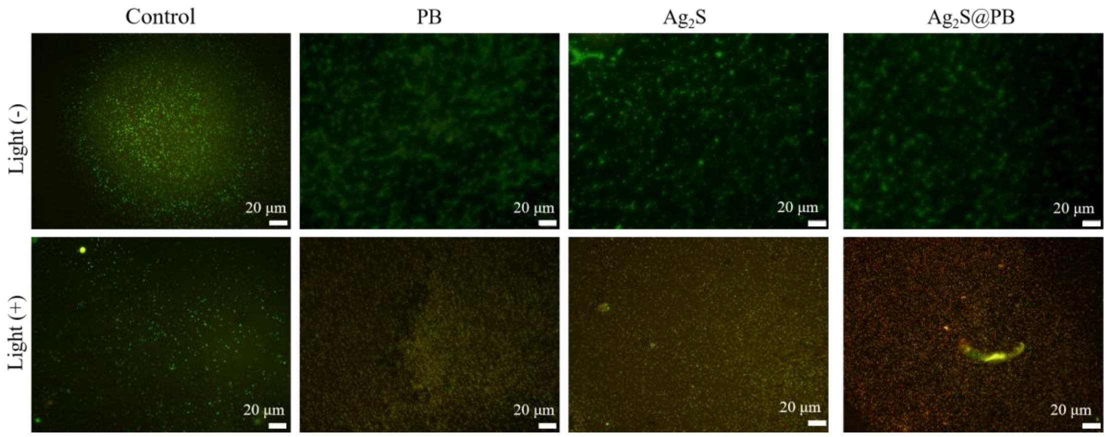

Figure S12. Bacterial live/dead staining. (Scale bars = 20 μm).

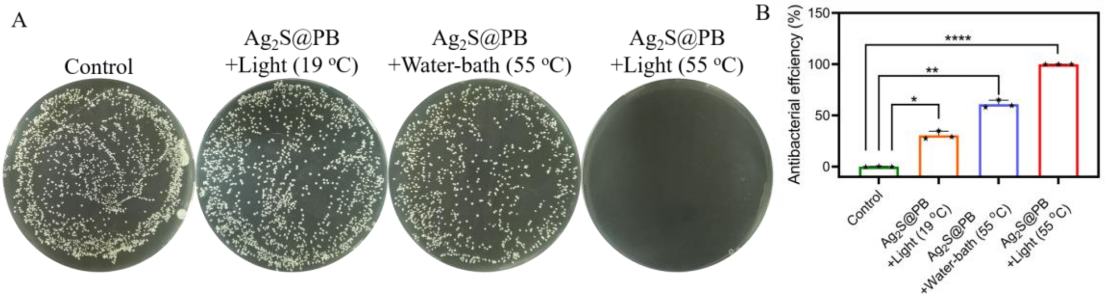

Figure S13. The influence of hyperthermia or ROS alone on antibacterial performance of Ag_2S@PB. (A) Images of Ag_2S@PB against *S. aureus* under different conditions. (B) The calculated germicidal efficiencies towards *S. aureus* in accordance with the spread plate counts. The error bars stand for means ± SD, n = 3. ns > 0.05, *p < 0.05, **p < 0.01, ***p < 0.001, ****p < 0.0001.

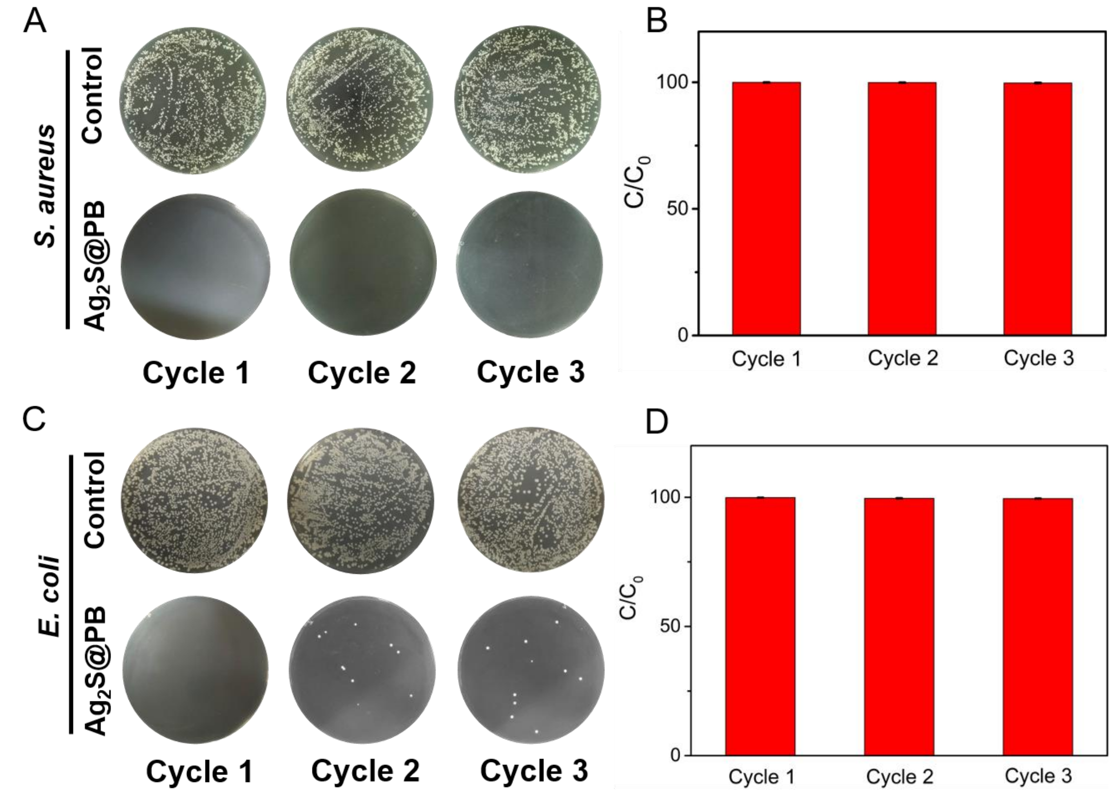

Figure S14. The spread plate outcomes against (A) *S. aureus* and (C) *E. coli* treated using Ag_2S@PB within three cycles of antimicrobial tests, respectively; The corresponding antimicrobial ratio histogram of (B) *S. aureus* and (D) *E. coli*. The error bars stand for means ± SD, n = 3.

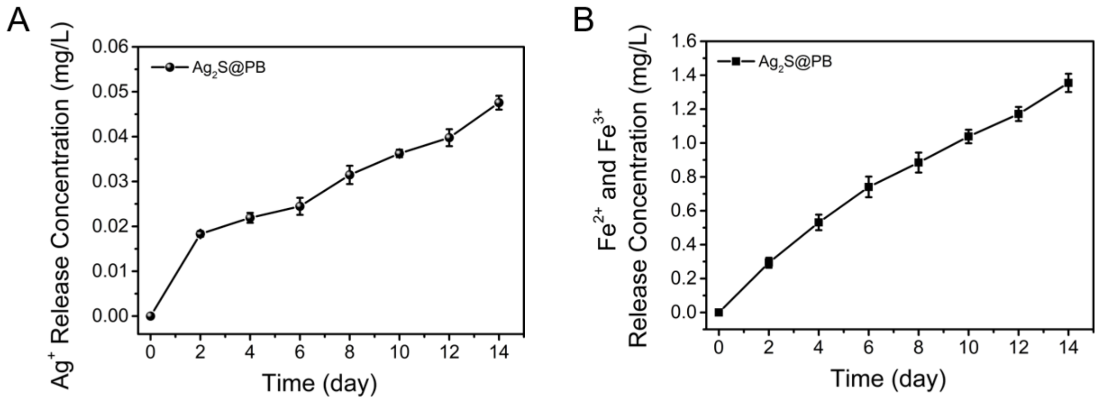

Figure S15. The ion release concentration from Ag_2S@PB. (A) Ag ions and (B) Fe ions. The error bars stand for means ± SD, n = 3.

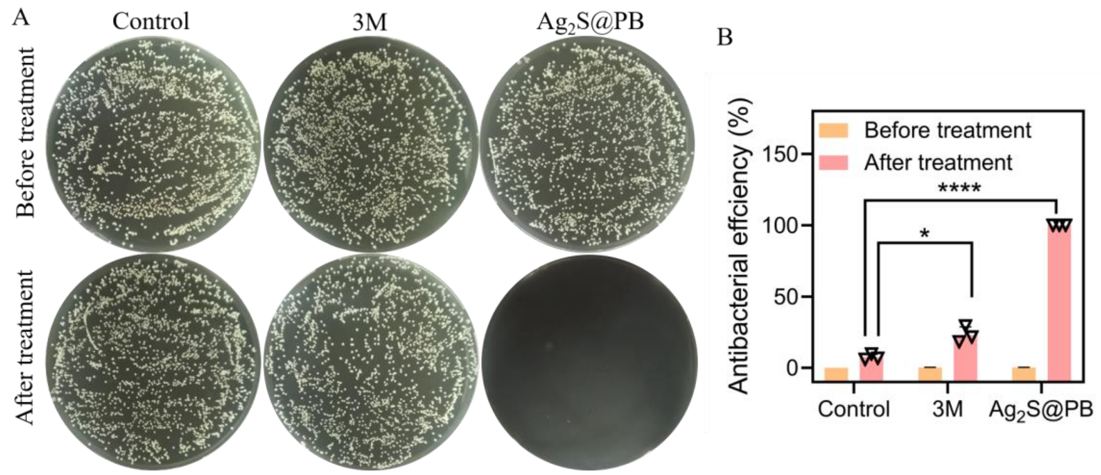

Figure S16. (A) Images of plate coating results before and after treatment of wound tissue on the back of mice. (B) The calculated germicidal efficiencies towards *S. aureus* in accordance with the spread plate counts. The error bars stand for means ± SD, n = 3. ns > 0.05, *p < 0.05, **p < 0.01, ***p < 0.001, ****p < 0.0001.

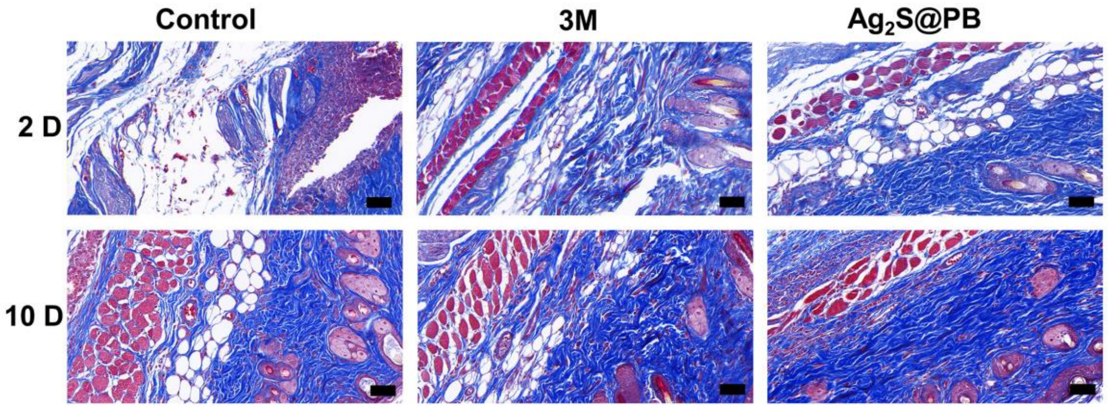

Figure S17. The Masson staining of Control, 3M, and Ag_2S@PB on 2 and 10 days. Scale bar = 50 μm.
